# From Transparency to Transport: Optoelectronic and Interfacial Signatures of n-Type ITO, FTO, ZnO and TiO_2_ Semiconductors

**DOI:** 10.3390/molecules31162868

**Published:** 2026-08-17

**Authors:** Júlia Holtz, Beatriz Moura Gomes, Vera C. M. Duarte, Joana Figueira, Joana Vaz Pinto, Luísa Andrade, Maria Helena Braga

**Affiliations:** 1 Faculty of Engineering, University of Porto, Rua Dr. Roberto Frias, 4200-465 Porto, Portugal; up201902282@up.pt (J.H.); up201805007@up.pt (B.M.G.); 2 MatER, Materials for Energy Research Laboratory, University of Porto, Rua Dr. Roberto Frias, 4200-465 Porto, Portugal; 3 LAETA, Associated Laboratory of Energy, Transports and Aerospace, Rua Dr. Roberto Frias, 400, 4200-465 Porto, Portugal; 4 LEPABE—Laboratory for Process Engineering, Environment, Biotechnology and Energy, Faculty of Engineering, University of Porto, Rua Dr. Roberto Frias, 4200-465 Porto, Portugal; veraduarte@fe.up.pt (V.C.M.D.); landrade@fe.up.pt (L.A.); 5 ALiCE—Associate Laboratory in Chemical Engineering, Faculty of Engineering, University of Porto, Rua Dr. Roberto Frias, 4200-465 Porto, Portugal; 6 Centro de Investigação de Materiais—CENIMAT|i3N, Department of Materials Science, School of Science and Technology, NOVA University Lisbon and Centro de Excelência de Microelectrónica e Optoelectrónica de Processos—CEMOP/UNINOVA, Campus de Caparica, 2829-516 Caparica, Portugal; j.figueira@fct.unl.pt (J.F.); jdvp@fct.unl.pt (J.V.P.)

**Keywords:** transparent conductive oxides, electron transport layers, ITO, FTO, ZnO, TiO_2_

## Abstract

Transparent conducting oxides and electron transport layers are central to optoelectronic devices, yet their interfacial electronic behavior remains strongly dependent on substrate chemistry, defect states, and surface potential alignment. Here, we compare ITO and FTO transparent electrodes coated with ZnO and TiO_2_, combining ab initio simulations, surface potential mapping, Hall effect measurements, sheet resistance, microscopy, and optical spectroscopy. Density functional calculations show that both ITO and FTO behave as degenerately doped n-type transparent conducting oxides, but with distinct work functions, surface dipoles, and donor-state distributions, leading to different interfacial charge-transfer tendencies. ZnO- and TiO_2_-coated substrates display markedly different temperature-dependent transport, including resistance hysteresis and carrier-type switching, with FTO-based heterojunctions showing more clearly defined transitions due to the greater thermal stability of FTO. Scanning Kelvin probe (SKP) measurements reveal that ZnO more effectively accepts electrons from ITO or FTO, whereas TiO_2_ shows weaker electron accumulation and more resistive interfacial behavior. Optical measurements and HSE06-based simulations confirm that TiO_2_ behaves as a wider-gap ultraviolet absorber, while ZnO exhibits a lower-energy absorption onset, with real spectra additionally shaped by substrate, thickness, scattering, and defect contributions. The results show that transparent conducting oxide substrates are active electronic participants, not passive supports, in ZnO- and TiO_2_-based optoelectronic interfaces.

## 1. Introduction

The integration of photovoltaic conversion and electrochemical storage into a single multifunctional device has attracted increasing attention as a promising candidate for next-generation energy devices [1]. Metal oxides play a central role, being widely employed as electrodes, transparent conductive oxides (TCOs), electron transport layers (ETLs), and interfacial modifiers, where their optoelectronic properties influence charge separation, transport, and recombination dynamics [2,3,4].

Indium tin oxide (ITO) and fluorine tin oxide (FTO) are TCOs that combine high electrical conductivity with optical transparency in the visible range between 80% and 95% [5,6,7,8] (Table 1), making them suitable as front electrodes in photovoltaics. Their transparency enables light to reach the photoactive material and help collect the carriers generated in the device [2]. ITO remains the most commonly used TCO; it is an n-type semiconductor with high carrier concentration ($10^{20}{\;\mathrm{cm}}^{-3}$ [9]) and low electrical resistivity ($10^{-3}–10^{-4}\;\Omega \cdot \mathrm{cm}$ [9,10]), alongside with its high optical transparency (ranging from 80% to 95% [5,6] Table 1) in the visible range and high electron mobility ($36–47\;{\mathrm{cm}}^{2}\;V^{-1}\;s^{-1}$ at 300 K [11]). However, its lower temperature stability (~200–350 °C [12,13]) and intrinsic brittleness limit its application, mainly in flexible devices [2,14]. To mitigate this limitation, flexible polymer substrates such as polyethylene terephthalate (PET) and polyethylene naphthalate (PEN) have been employed, enabling the fabrication of lightweight and bendable devices, although challenges related to mechanical stability and electrode integrity remain [2,14,15]. FTO typically behaves as an n-type semiconductor, exhibiting transmittance values in the range of 75–90% [8] across the visible spectrum depending on the temperature of the processing technique, making it particularly suitable as a front electrode in photovoltaic devices [8,16], showing lower electron mobility ($0.65–28.5\;{\mathrm{cm}}^{2}\;V^{-1}\;s^{-1}$ [17]) than ITO with a similar carrier concentration ($10^{20}\;{\mathrm{cm}}^{-3}$ [17]) and low electrical resistivity ($10^{-4}\;\Omega \cdot \mathrm{cm}$ [18]). Its wide optical bandgap (Table 1) ensures minimal absorption of visible light, allowing efficient transmission to the underlying photoactive layer [8]. Additionally, FTO films demonstrate good chemical and thermal stability, withstanding temperatures of up to 600–700 °C [18], which is essential for device durability under operating conditions and high-temperature applications [8,16].

Titanium oxide, TiO_2_, is widely used across diverse fields, ranging from biomedical applications and UV protection to photocatalysts and photodetectors [19,20,21,22]. As a transition metal oxide, TiO_2_ can exhibit multiple oxidation states, particularly $Ti^{3+}$ and $Ti^{4+}$, which give rise to tunable optical and electrical properties [23]. In the photovoltaic field, TiO_2_ is one of the most widely used ETLs for perovskite cells due to its suitable band alignment, chemical stability, and well-established processing methods [2,4]. It exhibits a wide bandgap (3.0–3.2 eV [4,21], Table 1), with a conduction band minimum of ~−4.1 eV [4] and valence band maximum of ~−7.2 eV [4], considering the absolute scale where the electrons at rest in a vacuum surface have zero energy, ensuring high transmittance in the visible region with electron mobility typically ranging from $10^{-5}$ to $10^{-4}\;{\mathrm{cm}}^{2}\;V^{-1}\;s^{-1}$ [24] and charge carrier density of $\sim 10^{17}\;{\mathrm{cm}}^{-3}$ [25]. However, its high annealing temperature (~500 °C [4]), interfacial energy barriers at the perovskite/$TiO_{2}$ contact and UV-induced defects formation can affect the device efficiency, stability, and cost [3,26].

Zinc oxide (ZnO) is being proposed as an alternative ETL for perovskite solar cells, given it is an n-type semiconductor with high electron mobility of $\sim 300\;{\mathrm{cm}}^{2}\;V^{-1}\;s^{-1}$ [25], suitable energy band alignment and bandgap of ~3.2–3.4 eV (Table 1) [25,27], high carrier density of $10^{17}–10^{22}$ ${\mathrm{cm}}^{-3}$ [28,29] and lower temperature (~150 °C [4]) processability, enabling cost-effective and flexible device fabrication [4,25,26,27,30]. Nevertheless, interfacial instability arising from surface defects and hydroxyl groups (−OH) at the perovskite/ZnO interface can trigger decomposition, enhance recombination losses and limit device efficiency and long-term stability [4,25]. Beyond its role as an ETL, the interfacial behavior of ZnO has also been investigated in metal/ZnO heterojunctions using SKP measurements paired with Density Functional Theory (DFT) calculations [31]. This approach enabled the mapping of surface chemical potentials, work function differences, and interfacial charge accumulation, suggesting a possible electrochemical-potential equalization via electrical double-layer capacitor formation at ZnO/metal interfaces [31].

This study focuses on a comparative analysis of TCOs, specifically ITO and FTO, which are widely employed as front electrodes in photovoltaic devices and other optoelectronic applications [8,16,32,33,34,35]. Despite sharing the common role of transparent conductors, these materials exhibit distinct electronic structures, carrier transport mechanisms, and surface properties, which critically influence their interfacial behavior with adjacent layers [32,36]. In particular, the TCO/ETL interface plays a key role in charge extraction, energy-level alignment, and recombination processes [34,35]. Their differences in parameters such as work function, carrier concentration, and surface morphology provide valuable insight into how these interfaces impact overall device performance [36,37,38]. Building on previous studies in which scanning Kelvin probe microscopy (SKPM) combined with DFT calculations was successfully applied to investigate work functions, surface chemical potentials, and interfacial charge redistribution in $TiO_{2}/metal$ interfaces [39], this work combines DFT and ab initio simulations with SKPM, SKP, the Hall effect, sheet resistance, UV–Vis spectroscopy, scanning electron microscopy (SEM), and atomic force microscopy (AFM) to provide a comprehensive characterization of ITO/FTO electrodes and their interfaces with TiO_2_ and ZnO layers.

**Table 1:** Comparative overview of different TCO and ETL properties.

Material Class	Specific Material	Key Properties	Role in the Device	Limitations	Chemical Potential (eV)	Bandgap (eV)	Ref.
**TCO**	ITO	High conductivity, up to 95% transmittance	Front Electrode	Brittle, poor flexibility	−4.2 to −5.0	3.93 to 4.35	[5,7,40,41,42]
	FTO	Suitable bandgap and transmittance for PV application	Front Electrode	Lower conduction then ITO Lower transmittance (75–90%)	−4.4	3.86 3.82 to 3.90	[8,16,43]
**ETL**	$TiO_{2}$	Wide bandgap, chemical stability, suitable band alignment	Facilitate carrier extraction	UV instability, interfacial recombination losses	−3.61 to −6.76 −4.4 −5.0	3.2 to 3.8 (anatase) 3.0 to 3.2 (rutile)	[4,21,44,45]
	ZnO	High electron mobility, low-temperature processing	Facilitate carrier extraction	Surface defects, chemical instability with perovskites (−OH groups)	−4.14 −4.55 to −4.56	3.2 to 3.4	[4,25,46,47]

## 2. Results and Discussion

**Figure 1:**
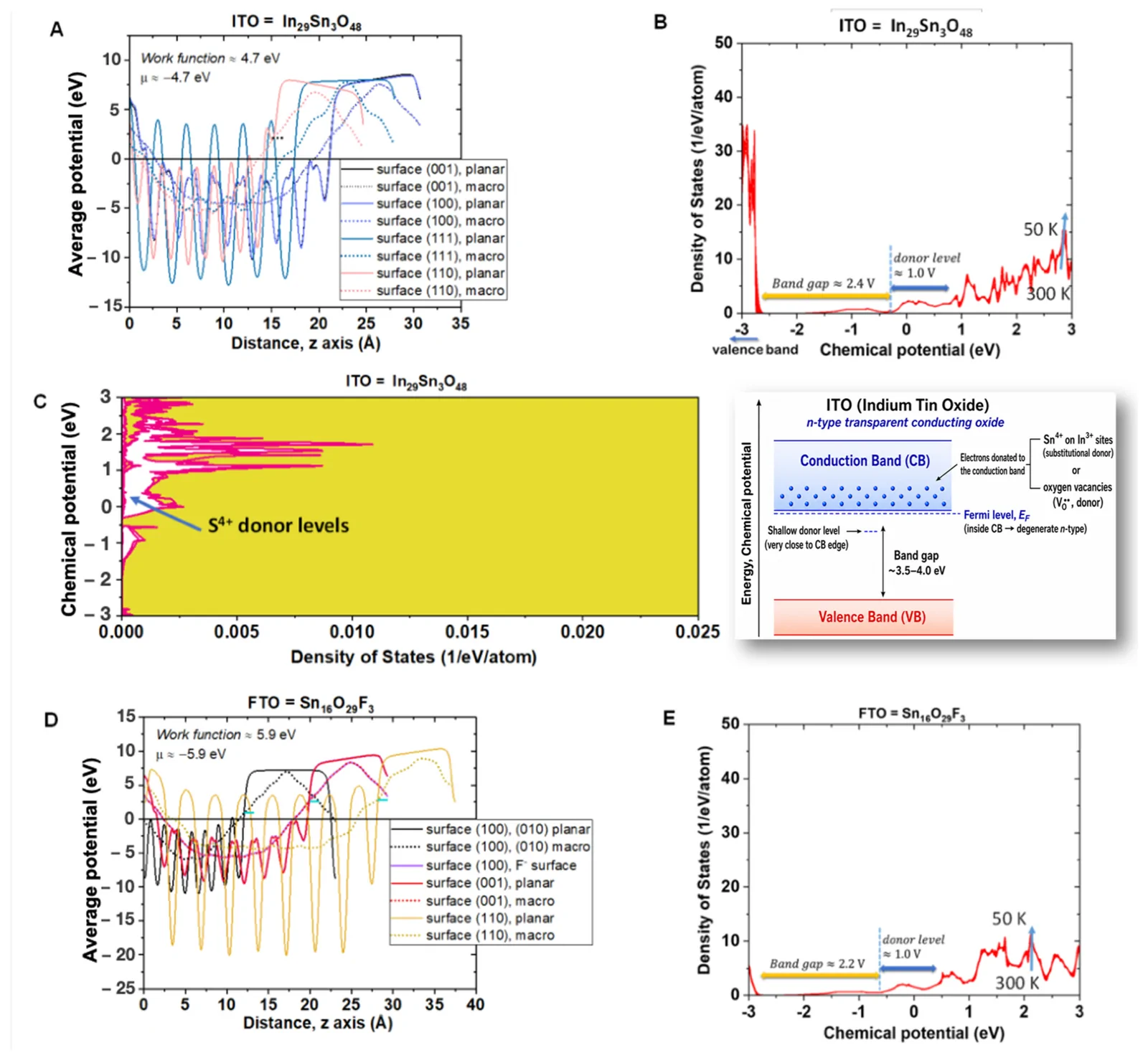
Electronic structure and work function comparison of ITO and FTO; (**A**) planar- and macroscopically averaged electrostatic potentials calculated along the surface-normal direction for ITO, In_29_Sn_3_O_48_, considering the (001)/(100), (110) and (111) surfaces. The vacuum-level alignment gives a work function of approximately 4.7 eV, with the electronic chemical potential located at μ ≈ −4.7 eV when the surface containing a Sn^4+^ is considered in (110) (dashed mark); (**B**) Total density of states of ITO, normalized per atom, as a function of chemical potential. The calculated valence to conduction band separation is approximately 2.4 eV, while Sn-derived donor states appear close to the conduction band edge, within an energy range of about 1.0 eV; (**C**) (**left**) enlarged low-DOS representation for ITO, highlighting the Sn-related donor states close to the Fermi level and (**right**) schematic band structure for ITO illustrating its degenerate n-type character, in which substitutional Sn^4+^ on In^3+^ sites and oxygen vacancies donate electrons to the conduction band; (**D**) planar- and macroscopically averaged electrostatic potentials for FTO, Sn_16_O_29_F_3_, considering the (100)/(010), (001), and (110) surfaces and the (100) surface containing a F^−^ at the surface. The resulting vacuum-level alignment yields a work function of approximately 5.9 eV, corresponding to μ ≈ −5.9 eV; however, as for ITO, the work function may be concentration dopant dependent; (**E**) Total density of states of FTO, normalized per atom. The calculated valence to conduction band separation is approximately 2.2 eV, with F-induced donor states located close to the conduction band edge over an energy interval of about 1.0 eV. In both materials, the finite density of states near the Fermi level is consistent with degenerate n-type semiconductor conductivity.

By using ab initio simulations, we have calculated the electrochemical potential, work function and density of states of ITO and FTO, Figure 1. The calculated surface electrostatic potentials and electronic density of states show differences between ITO (In_29_Sn_3_O_48_), and FTO (Sn_16_O_29_F_3_), despite both behaving as degenerately doped n-type transparent semiconductor oxides, Figure 1.

For ITO, the planar averaged potential exhibits pronounced oscillations across the slab, reflecting the atomic-scale variation in the ionic and electronic charge densities. The macroscopically averaged potential smooths these oscillations and allows the vacuum level to be identified. From the vacuum alignment, a work function of approximately 4.7 eV is obtained, corresponding to an electronic chemical potential of µ ≈ −4.7 eV relative to electrons at rest in a vacuum, Figure 1A,C. The (001) and (100) surfaces yield broadly similar vacuum levels, although the microscopic potential profiles may differ due to their distinct atomic stacking and terminations, as evidenced by the ITO calculations presented in Figure 1A, where the most reliable simulated work function is obtained; consequently, chemical potential is the one given by the stack containing a surface dopant (Sn^4+^). The work function is determined not only by the bulk Fermi level but also by the surface dipole associated with each crystallographic orientation.

FTO exhibits a higher calculated work function of approximately 5.9 eV, corresponding to µ ≈ −5.9 eV. The (100)/(010), (001), and (110) surfaces show visibly different planar and macroscopic potential profiles, indicating a stronger dependence on facet and surface termination, Figure 1D. This behavior is consistent with the rutile SnO_2_ structure, in which the coordination and density of exposed Sn, O, and F species vary significantly between surfaces. The approximately 1.2 eV difference between the work functions of FTO and ITO places the FTO Fermi level at a deeper absolute energy and may reflect an inadequate positioning of the F^−^ dopant regarding the surface, in agreement with similar verification for ITO and experimental data. Consequently, the two transparent electrodes are expected to establish different interfacial potential barriers and charge-transfer driving forces when contacted with ZnO or TiO_2_.

The total density of states of ITO gives a calculated valence to conduction band separation of approximately 2.4 eV (Figure 1B,C). This is lower than the commonly reported experimental optical gap of ITO, as expected from the underestimation of semiconductor bandgaps by semilocal density functional approximations. Nevertheless, the calculation reproduces the essential characteristics of the doped oxide. Weak Sn-derived donor states appear close to the conduction band edge, extending over an energy interval of approximately 1.0 eV. These states are more clearly visible in the enlarged low electronic density of states (DOS) representation (Figure 1B,C).

Substitutional Sn^4+^ on In^3+^ sites contributes one excess electron per dopant, shifting the Fermi level toward or into the In (5s)-derived conduction band (Figure 1C). Oxygen vacancies may provide additional donor contributions depending on their local environment and charge state. The finite density of states close to the Fermi level is therefore consistent with the degenerate n-type character of ITO. In this regime, the lowest conduction band states are partially occupied, which may also shift the apparent optical absorption edge to higher energies through the Burstein–Moss effect. Oxygen vacancies are discussed here only as a possible additional donor mechanism in experimental ITO; they were not included in the In_29_Sn_3_O_48_ computational model used in this work.

The widely used 90 wt.% In_2_O_3_/10 wt.% SnO_2_ ITO formulation corresponds to approximately 9.3 at.% Sn on the cation sublattice, closely matching the 9.4 at.% represented by In_29_Sn_3_O_48_ and is, therefore, used herein for simulations of the ITO.

A similar electronic structure was obtained for FTO. The calculated valence to conduction band separation is approximately 2.2 eV, again lower than the experimental optical gap generally reported for fluorine-doped SnO_2_ (Figure 1E). Fluorine substitution on oxygen sites, $F_{O^{2-}}^{-}$, provides one excess electron per substitution and generates donor-related states close to the Sn (5s)-dominated conduction band edge. These donor states extend over an interval of approximately 1.0 eV, while the finite DOS around the Fermi level confirms that the simulated FTO composition is also degenerately n-type (Figure 1E).

Although the calculated electronic gaps of ITO and FTO are similar, their absolute energy-level positions differ. FTO combines a deeper chemical potential and larger work function with a donor-rich conduction band region, whereas ITO displays a shallower chemical potential and lower work function. This distinction is highly relevant for transparent-electrode/electron-transport-layer heterojunctions. The lower work function of ITO should favor electron donation to an adjacent electron-accepting oxide more strongly than FTO, although the final interfacial charge redistribution will also depend on the electron affinity, surface defects, morphology, and chemical potential of the deposited ZnO or TiO_2_ layer.

The DOS and surface potential energy calculations are therefore complementary. The DOS describes the distribution of available electronic states relative to the Fermi level, whereas the vacuum-referenced surface calculations establish their absolute energetic position. Together, the results show that ITO and FTO are both degenerate n-type transparent conducting oxides, but they are not electronically equivalent. Their distinct work functions, facet-dependent surface dipoles, and donor-state distributions are expected to produce different band alignment, charge accumulation, and transport behavior at the corresponding oxide interfaces.

**Figure 2:**
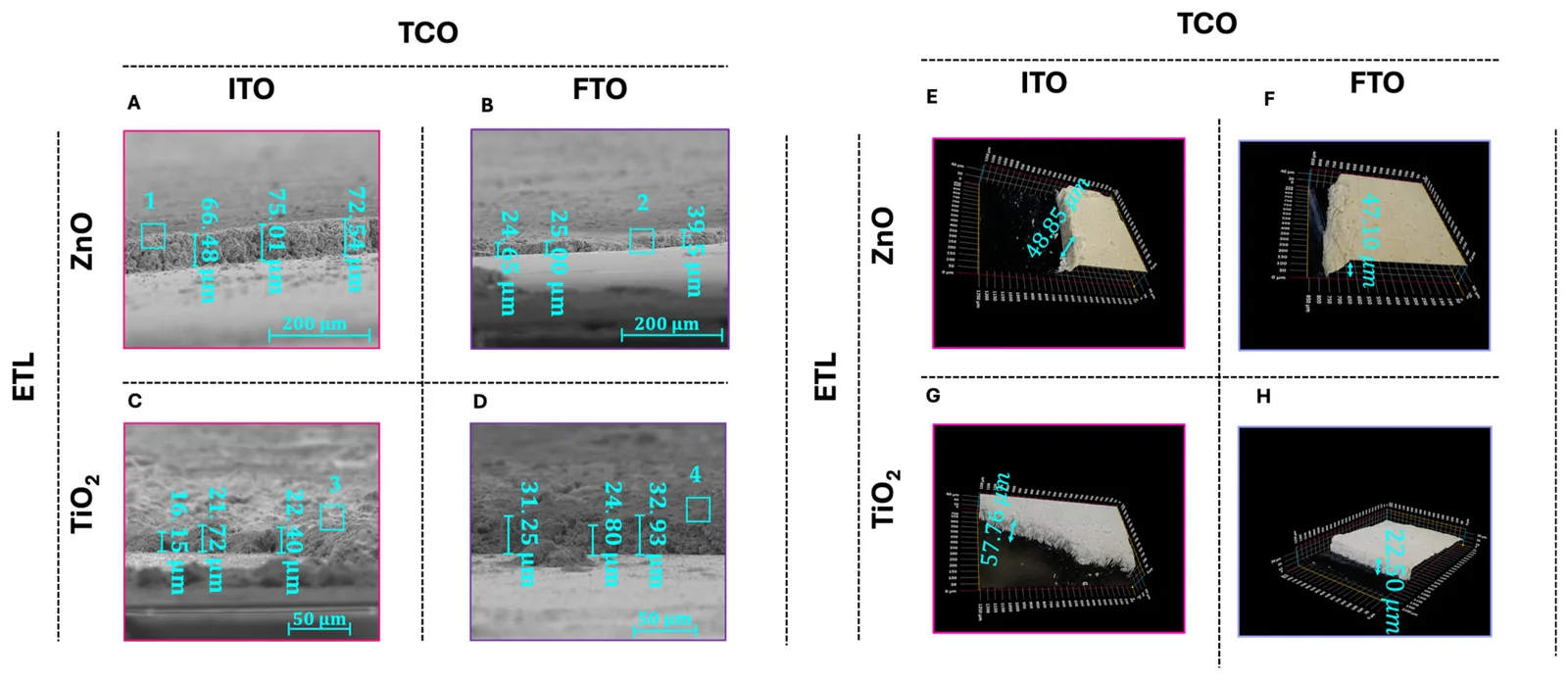
Imaging of substrate and deposited materials. SEM-acquired images with secondary electron detector of (**A**) ZnO@ITO, (**B**) ZnO@FTO, (**C**) TiO_2_@ITO and (**D**) TiO_2_@FTO at magnification of 500× for the ZnO@TCO and 1200× for the TiO_2_@TCO. Motorized digital microscope imaging 3D surface reconstruction of (**E**) ZnO@ITO, (**F**) ZnO@FTO, (**G**) TiO_2_@ITO and (**H**) TiO_2_@FTO at objective of 20×. Note: ETL—electron transport layer; TCO—transparent conductive oxide. Average thicknesses—SEM: ZnO@ITO: 65 µm; ZnO@FTO: 34 µm; TiO_2_@ITO: 29 µm; TiO_2_@FTO: 28 µm.

To investigate the influence of ETL on TCOs, ZnO and TiO_2_ were deposited onto ITO and FTO substrates by drop coating. SEM imaging of the bare substrates, prior to ETL deposition, can be found in Figure S1A,B, Supplementary Information. Since the thickness of the ETL strongly influences the optical and electrical performance of the final device, the deposited layers were characterized by SEM, Figure 2A–D, and surface reconstruction using a motorized digital microscope, Figure 2E–H and Figure S1C–F, Supplementary Information. For comparison, an additional flexible PET-ITO substrate was also characterized and is presented in Figure S2, Supplementary Information.

In both cases, ZnO-coated and TiO_2_-coated TCOs show comparable layer thicknesses (between 20 and 75 μm, Figure 2 and Figure S2B–E, Supplementary Information), although the TiO_2_ coatings were consistently thinner than the ZnO coatings. Furthermore, the SEM images reveal significant differences in the morphology of the deposited layers. The ZnO coating (Figure 2A,B) exhibits a denser, more compact and robust microstructure, whereas the TiO_2_ layer (Figure 2C,D) appears less consolidated, presenting a rougher surface morphology and a less homogeneous structure. Energy-dispersive X-ray spectroscopy (EDX) analysis of the deposited oxide layers confirmed the absence of detectable contamination (Table S1, Supplementary Information).

**Figure 3:**
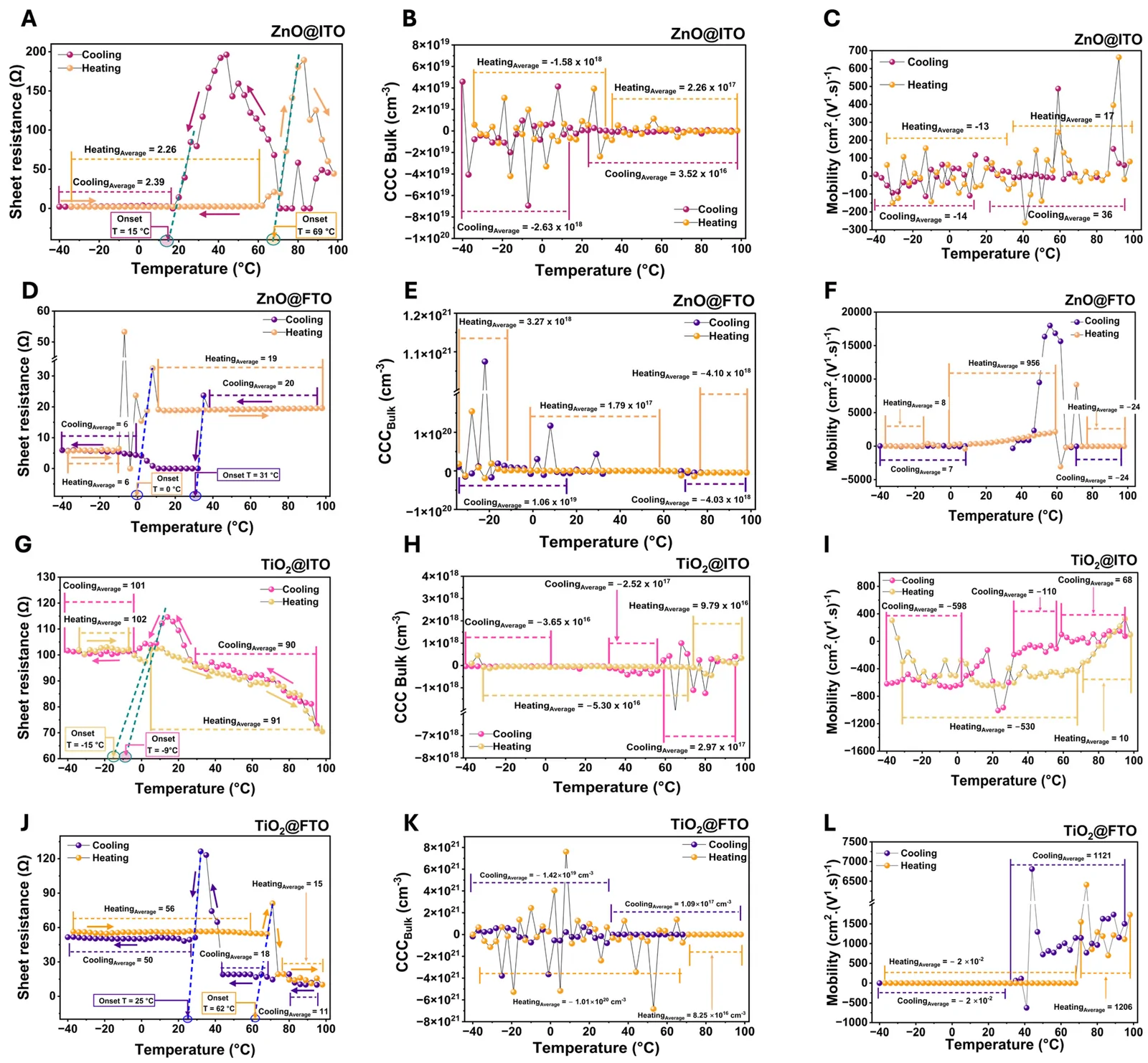
Electrical characterization. Sheet resistance, charge carrier concentration and mobility vs. temperature curves of ZnO@ITO (**A**–**C**); ZnO@FTO (**D**–**F**); TiO_2_@ITO (**G**–**I**) and TiO_2_@FTO (**J**–**L**), respectively. Note: samples’ surface area 6.25 cm^2^; ITO and FTO film thicknesses: 120–160 nm and 320–340 nm, respectively, and thicknesses of the ETLs: ZnO@ITO: 65 µm; ZnO@FTO: 34 µm; TiO_2_@ITO: 29 µm; TiO_2_@FTO: 28 µm.

Sheet resistance and Hall effect measurements were performed on the ETL-coated TCOs using a four-point gold probe configuration. From these measurements, the sheet resistance, charge carrier concentration, and carrier mobility were determined for all samples (Figure 3, Figures S3 and S4, Supplementary Information). ITO and FTO exhibited the expected behavior of conductive materials, with sheet resistance increasing as the temperature increased (ITO: Figure S3A–C and FTO: Figure S3D–F, Supplementary Information). The average sheet resistance calculated over the temperature range from −40 to 100 °C was approximately 8 Ω/0.12 µm ≈ 67 × 10^6^ Ωm^−1^ for ITO during both heating and cooling (Figure S3A, Supplementary Information) and 19 Ω/0.330 µm ≈ 58 × 10^6^ Ωm^−1^ for FTO, Figure S3D, Supplementary Information. These values are consistent with the nominal sheet resistance specified by the manufacturer (ITO < 10 Ω and FTO ≈ 15 Ω. Manufacturer information in Section 3). The calculated charge carrier concentration was negative (e^−^) for both materials, corresponding to −5.68 × 10^20^ cm^−3^ for ITO during both heating and cooling (Figure S3B, Supplementary Information) and −4.05 × 10^20^ cm^−3^ for FTO during both heating and cooling, Figure S3E, Supplementary Information. These results lead measured mobility values, which were also negative for both substrates, as expected, averaging approximately −40 cm^2^ V^−1^ s^−1^ for ITO (Figure S3C, Supplementary Information) and −23 cm^2^ V^−1^ s^−1^ for FTO, Figure S3F, Supplementary Information.

The sheet resistance, charge carrier concentration, and carrier mobility of ZnO-coated ITO are presented in Figure 3A–C, respectively. During heating, the average sheet resistance remained approximately constant at 2.26 Ω between −40 and 60 °C, Figure 3A. Since ZnO is a wide-bandgap semiconductor, the dependence of sheet resistance on temperature contrasts with that of the bare TCO substrate (a conductor—with linear dependency between sheet resistance and temperature, Figure S3A, Supplementary information). At 69 °C, a phase transition induces a sudden shift from a low- to a high-resistance state (Figure 3A). The sheet resistance reaches a maximum of 200 Ω at 80 °C, subsequently decaying to a value of roughly 50 Ω around 100 °C (Figure 3A). During cooling, the sheet resistance abruptly increases at 71 °C, reaching a peak of 200 Ω at 45 °C (Figure 3A). Below 45 °C, the resistance drops sharply, stabilizing at an average of 2.39 Ω between 15 °C and −40 °C (Figure 3A). The charge carrier concentration and mobility exhibited two distinct temperature regimes during both heating and cooling. During heating, from −40 to 25 °C, the average charge carrier concentration was −1.58 × 10^18^ cm^−3^, with a corresponding mobility of −13 cm^2^ V^−1^ s^−1^, Figure 3B,C, confirming n-type conduction dominated by e^−^. Above this interval (35 to 100 °C), the average charge carrier concentration changed to 2.26 × 10^17^ cm^−3^, while the average mobility increased to 17 cm^2^ V^−1^ s^−1^, confirming p-type conduction dominated by h^+^. A similar behavior was observed during cooling. From 100 to 30 °C, the average charge carrier concentration and mobility were 3.52 × 10^16^ cm^−3^ and 36 cm^2^ V^−1^ s^−1^, respectively, whereas from 10 to −40 °C, the values changed to −2.63 × 10^18^ cm^−3^ and −14 cm^2^ V^−1^ s^−1^, Figure 3B,C.

In contrast, ZnO-coated FTO (Figure 3D–F) exhibited a significantly different temperature-dependent response. Compared with its ITO-based counterpart (Figure 3A), the FTO-based sample exhibited a much more pronounced sheet resistance hysteresis (Figure 3D). The hysteresis observed for the ZnO-coated ITO sample was less pronounced, consisting of sharp resistance peaks rather than a well-defined hysteresis loop (Figure 3A). A similarly less defined hysteresis was also observed for the ZnO-coated PET/ITO sample (Figure S3J, Supplementary Information). These differences can be attributed to the greater thermal stability of the FTO substrate relative to ITO and PET/ITO, which allows the temperature-dependent electrical response of the ZnO@FTO interface to remain more clearly distinguishable throughout the heating and cooling cycles.

The ZnO@FTO system exhibited two distinct transport regimes separated by a clear thermal hysteresis (Figure 3D). During heating, the sheet resistance remained near 6 Ω between −40 and −10 °C, increased abruptly around 0 °C, and stabilized at approximately 19 Ω from 10 to 100 °C (Figure 3D). During cooling, it remained close to 20 Ω down to 35 °C before decreasing sharply near 31 °C and returning to approximately 6 Ω below 0 °C (Figure 3D). Hall measurements confirmed corresponding changes in the dominant carriers (Figure 3E,F). During heating, hole-dominated conduction was observed below 60 °C, followed by a transition to electron-dominated transport above 75 °C (Figure 3E,F). During cooling, electron-dominated conduction persisted from 100 to 70 °C, while a pronounced mobility peak appeared between 65 and 45 °C without a comparable increase in carrier concentration (Figure 3E,F). Below 5 °C, hole-dominated transport was recovered.

For the ZnO-coated TCO substrates, the resistance of the heterojunction decreases at low temperatures (Figure 3A,E and Figure S3M, Supplementary Information). This behavior is related to the greater tendency of ZnO to accept electrons from the TCO. The TCO ZnO-coated heterojunction exhibits a more conductor-like temperature dependence, characterized by higher resistance at elevated temperatures and lower resistance at reduced temperatures (Figure 3A,E and Figure S3M, Supplementary Information). The resulting charge transfer from the TCO to the ZnO layer increases the electron concentration in the latter, while simultaneously modifying the electrical properties of the ZnO@TCO interface. This process may also be associated with an increased concentration of oxygen vacancies and/or the formation of a Zn-containing alloy or interfacial phase.

A possible microscopic origin of the apparent carrier-type reversal is the temperature-dependent redistribution and charge-state evolution of native and surface defects. In ZnO, oxygen vacancies and Zn-related donor defects may contribute electron states, whereas chemisorbed oxygen and surface hydroxyl species can trap or release carriers as temperature changes. Their relative populations and ionization states can therefore shift the balance of the Hall response and modify the effective interfacial carrier density. In TiO_2_, thermally activated oxygen-vacancy states provide an analogous donor contribution. Because these processes are coupled to adsorption/desorption and interfacial charge redistribution, they may also produce path-dependent behavior during heating and cooling. This mechanism is proposed as an interpretation of the Hall and resistance data rather than as direct identification of a specific defect species.

For the TiO_2_-coated ITO (Figure 3G–I), the average sheet resistance during heating was 102 Ω between −30 and −10 °C, followed by a phase transition at −15 °C. From 5 to 100 °C, the sheet resistance stabilized with an average value of 91 Ω, Figure 3G. During cooling, the average sheet resistance was 90 Ω between 100 and 30 °C, followed by a phase transition at −9 °C, Figure 3G. Between −10 and −40 °C, the sheet resistance stabilized with an average value of 101 Ω, Figure 3G.

During heating, the average charge carrier concentration between −30 and 70 °C was −5.3 × 10^16^ cm^−3^, confirming n-type semiconductor conduction dominated by e^−^, with a corresponding negative mobility of −530 cm^2^ V^−1^ s^−1^ (Figure 3H,I). Between 70 and 100 °C, the average charge carrier concentration was 9.69 × 10^16^ cm^−3^, confirming p-type semiconductor conduction dominated by h^+^, with a corresponding mobility of 10 cm^2^ V^−1^ s^−1^, Figure 3H,I. The average charge carrier concentration during cooling between 100 and 60 °C was 2.97 × 10^17^ cm^−3^, confirming p-type semiconductor conduction dominated by h^+^, with a corresponding positive mobility of 68 cm^2^ V^−1^ s^−1^, Figure 3H,I. From 60 to 30 °C, the average charge carrier concentration was −2.52 × 10^17^ cm^−3^, showing n-type semiconductor conduction dominated by e^−^, with a corresponding mobility of −110 cm^2^ V^−1^ s^−1^, Figure 3H,I. From 5 to −40 °C, the average charge carrier concentration was −3.65 × 10^16^ cm^−3^, with a corresponding mobility of −598 cm^2^ V^−1^ s^−1^, Figure 3H,I. For comparison, a TiO_2_-coated PET/ITO substrate was also characterized by sheet resistance and Hall effect measurements showing very similar behavior, Figure S3J–L.

Contrary to the behavior observed for the ZnO-coated substrates, all TiO_2_-coated substrates exhibited an increase in sheet resistance at low temperatures (Figure 3G,J and Figure S3J, Supplementary Information). This trend was consistently observed for the ITO- FTO-, and PET/ITO-based samples, despite the corresponding bare substrates exhibiting a decrease in resistance upon cooling (Figure S3A–I, Supplementary Information). Therefore, unlike the ZnO-coated systems, the temperature-dependent electrical response of the TiO_2_-coated heterojunctions is governed primarily by the TiO_2_ layer. As a non-degenerate semiconductor, TiO_2_ relies on thermally activated defects, such as oxygen vacancies, to donate electrons to the conduction band. Cooling suppresses this thermal activation, reducing carrier mobility and consequently increasing the resistivity (Figure 3G,J and Figure S3J, Supplementary Information). Similarly to the ZnO-coated substrates, the TiO_2_-coated FTO sample exhibited a more clearly defined sheet resistance hysteresis than the corresponding ITO- and PET/ITO-based samples, consistent with the greater thermal stability of the FTO substrate. During heating, the TiO_2_-coated FTO sheet resistance remained approximately 56 Ω from −40 to 60 °C, before decreasing sharply near 62 °C and reaching an average of 15 Ω, between 75 and 100 °C (Figure 3J). During cooling, values of approximately 11 and 18 Ω were obtained over the intervals of 100–80 °C and 70–45 °C, respectively. Below the transition near 25 °C, the resistance increased and stabilized at approximately 50 Ω down to −40 °C. The Hall effect results reveal a corresponding reversal in the dominant transport mechanism (Figure 3K,L). For the heating process, electron-dominated conduction prevailed between −40 and 65 °C, with an average carrier concentration of −1.01× 10^20^ cm^−3^ and mobility of −2 × 10^−2^ cm^2^ V^−1^ s^−1^. Above 70 °C, both parameters became positive, reaching 8.25 × 10^16^ cm^−3^ and 1206 cm^2^ V^−1^ s^−1^, respectively, indicating hole-dominated transport. For the cooling part, this p-type regime persisted from 100 to 30 °C, with average values of 1.09 × 10^17^ cm^−3^ and 1121 cm^2^ V^−1^ s^−1^. Below 25 °C, electron-dominated conduction was recovered, with a carrier concentration of −1.42 × 10^19^ cm^−3^ and mobility of −2 × 10^−2^ cm^2^ V^−1^ s^−1^ (Figure 3K,L).

To investigate the electrochemical behavior of these systems, the chemical potential of the materials was determined using μm-resolution scanning Kelvin probe (μm-SKP) measurements and nm-resolution SKPM. For the μm-resolution measurements, samples with a Cu/ETL-coated TCO/Al architecture were analyzed. The ETL-coated TCO region, approximately 5 mm in length, was exposed to the SKP tip, while the Cu electrode provided the electrical connection to the instrument. The scan was performed from the Cu electrode, across the ETL-coated TCO region, and toward the Al electrode. For the nm-resolution SKP measurements, a 20 × 20 μm^2^ area of the ETL-coated TCO surface was analyzed.

To establish a reference, the surface chemical potentials of bare ITO, FTO, and PET/ITO were firstly characterized using the same approach (Figure S5, Supplementary Information). The μm-resolution measurements were performed in a Cu/TCO/Al configuration, whereas the nm-resolution measurements were carried out directly on the bare TCO surfaces (Figure S5, Supplementary Information).

When incorporated into the Cu/ITO/Al configuration, electron transfer occurs from Al toward ITO and Cu (Figure S5A, Supplementary Information). The Cu electrode is electrically connected to the SKP, allowing electron transfer through Cu and from Cu to the SKP. In the 1st frame, the chemical potentials of ITO and Cu align at an average value of approximately −0.19 V vs. SHE (−4.25 eV), while the Al potential remains at −0.82 V vs. SHE (−3.62 eV) (Figure S5A, Supplementary Information). In the third frame, the ITO and Cu potentials align at approximately −0.05 V vs. SHE (−4.39 eV), approaching the chemical potential of bare ITO measured by nm-resolution SKP (−0.01 V vs. SHE, −4.43 eV, Figure S5B, Supplementary Information, in agreement with the literature as reported in Table 1), while the Al potential shifts to −0.60 V vs. SHE (−3.84 eV). These results indicate that electrons are transferred from Al through ITO toward Cu and subsequently from Cu to the SKP.

For the Cu/FTO/Al configuration, the Al potential remains at −0.65 V vs. SHE (−3.79 eV) across all frames. Meanwhile, the chemical potentials of FTO and Cu align at approximately 0.15 V vs. SHE (−4.59 eV) in the first measurement and 0.04 V vs. SHE (−4.48 eV) in the third (Figure S5C, Supplementary Information). In the nm-SKP measurements, bare FTO exhibits a potential of 0.30 V vs. SHE (−4.74 eV) (Figure S5D, Supplementary Information). Considering that Cu also has an individual potential of approximately 0.30 V vs. SHE (−4.74 eV) [31,39,48], their shift to 0.04 V vs. SHE (−4.48 eV) in the Cu/FTO/Al heterojunction indicates electron transfer from Al to Cu and FTO. The Al chemical potential shows periodic oscillations with a wavelength (λ) of approximately 1350 μm, Figure S5C Supplementary Information. A similar phenomenon has previously been reported for Al in a Cu/SiO/Al configuration, exhibiting a comparable oscillation wavelength [45]. FTO consists predominantly of F-doped SnO_2_ and therefore contains a substantially higher SnO_2_ fraction than ITO. Since Sn and Si belong to the same group of the periodic table and both SnO_2_ and SiO behave as n-type semiconductors, the similarity between these systems may account for the comparable oscillatory behavior. Furthermore, the metallic nature of Al, together with its high and flat electronic density of states above and below the Fermi level, enables efficient charge exchange with the adjacent semiconductor while accommodating a broad range of measured chemical potentials, from approximately −1 V vs. SHE (−3.44 eV) to +0.3 V vs. SHE (−4.74 eV) [31,39,48].

The Cu/PET/ITO/Al system exhibited a progressive shift in the PET/ITO chemical potential toward more negative values over successive measurements, reaching approximately −0.30 V vs. SHE (−4.14 eV) in the final measurement (Figure S5E, Supplementary Information). Electrons flow from Al through PET/ITO toward Cu, as indicated by the Al potential becoming considerably more positive than its expected value of approximately −1 V vs. SHE (−3.44 eV). However, the absence of potential alignment between PET/ITO and Cu suggests that electron transport from PET/ITO to the Cu electrode is limited. Consequently, electrons accumulate on the PET/ITO surface, shifting its potential from the bare value of 0.57 V vs. SHE (−5.01 eV) to increasingly negative values (Figure S5F, Supplementary Information). This behavior is associated with the higher effective resistance of the flexible conductive layer, as supported by the sheet resistance measurements (PET/ITO, ITO thickness: 800 Ω/0.200 µm = 4000 × 10^6^ Ωm^−1^, Figure S3G; ITO: 8 Ω/0.140 µm = 67 × 10^6^ Ωm^−1^, Figure S3A; and FTO: 58 × 10^6^ Ωm^−1^, Figure S3D, Supplementary Information) and coating discontinuities or inhomogeneities observed by SEM (Figure S2A, Supplementary Information).

**Figure 4:**
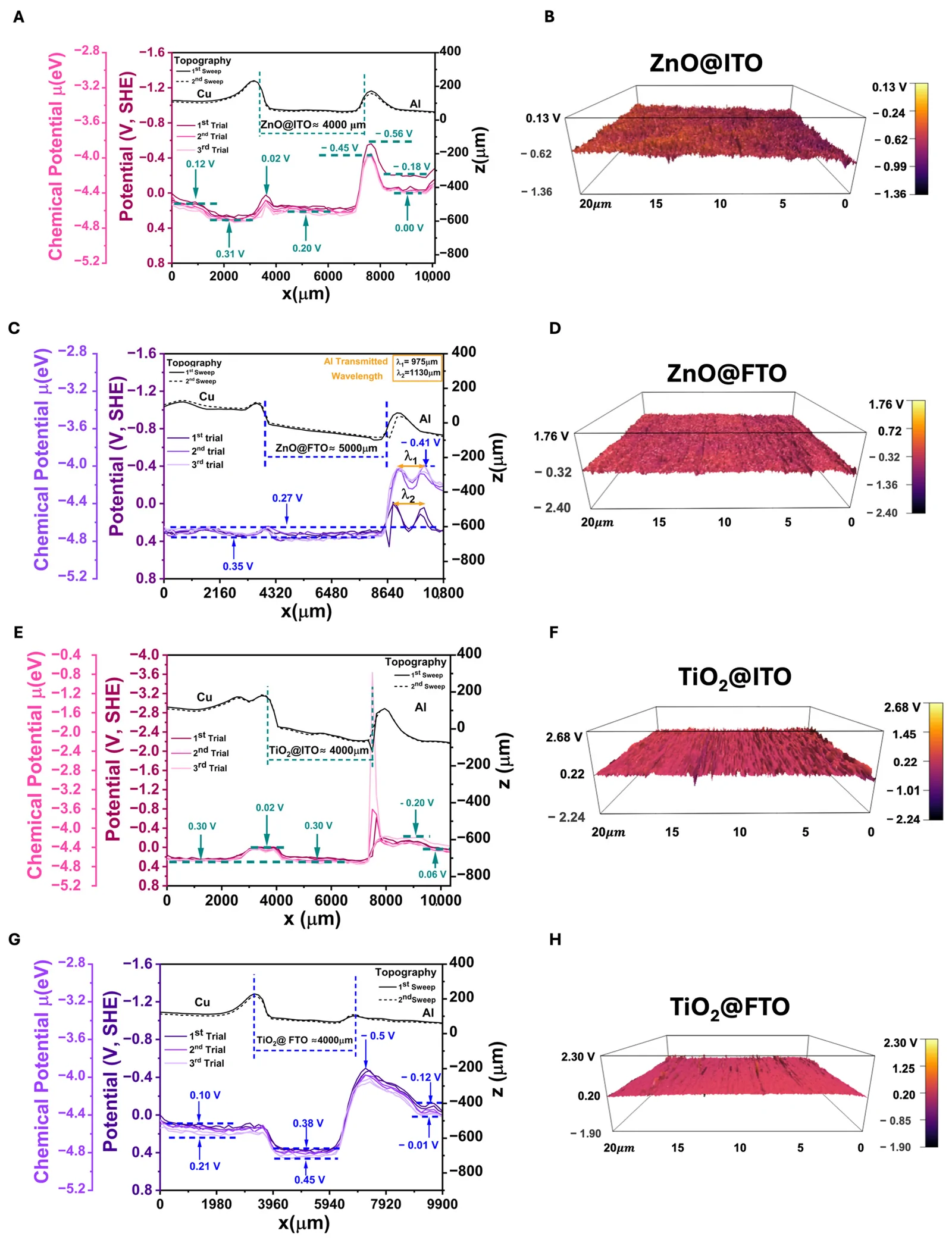
Electrochemical characterization. Micrometer-resolution SKP profile across Cu/ETL@TCO/Al setup, and nanometer-resolution SKPM (AFM potential retrace) of ETL@TCO surface, respectively, (**A**,**B**) ZnO@ITO, (**C**,**D**) ZnO@FTO, (**E**,**F**) TiO_2_@ITO and (**G**,**H**) TiO_2_@FTO.

In the Cu/ZnO@ITO/Al architecture, the Cu potential initially measures approximately 0.12 V vs. SHE (−4.56 eV), due to the electrical connection between the Cu electrode and the SKP system, which promotes charge exchange as the electrode and instrument approach potential equilibration (Figure 4A). The Cu chemical potential then stabilizes at approximately 0.31 V vs. SHE (−4.75 eV), consistent with the expected value for Cu chemical potential [31,39,48], Figure 4A. At nanometer resolution, ZnO@ITO displays a chemical potential of −0.62 V vs. SHE (−3.82 eV) (Figure 4B), substantially more negative than the values of bare ITO (0.30 V vs. SHE, −4.74 eV) and ZnO (0.40 V vs. SHE, −4.84 eV [31]). This shift suggests that contact between ZnO and ITO promotes electron transfer from ITO to ZnO, leading to electron accumulation and negative charging at the ZnO surface, when bare, Figure 4B. When the ZnO@ITO heterojunction is incorporated into the Cu/ZnO@ITO/Al architecture, electron tunneling occurs among the three components until their chemical potentials approach “equilibrium”. The resulting redistribution of charge explains the approximately 0.8 eV difference between the chemical potential of the isolated ZnO@ITO interface and that measured in the complete Cu/ZnO@ITO/Al architecture.

In the Cu/ZnO@ITO/Al configuration, additional charge redistribution occurs with ZnO@ITO exhibiting an average potential of approximately 0.20 V vs. SHE (−4.64 eV), Figure 4A. Distinct negative potential peaks are observed at both the Cu/ZnO@ITO and Al/ZnO@ITO interfaces, indicating localized electron accumulation, Figure 4A. The most pronounced accumulation occurs at the Al/ZnO@ITO interface, where the potential reaches −0.56 V vs. SHE (−3.88 eV) during the first measurement and stabilizes at approximately −0.45 V vs. SHE (−3.99 eV) in the second and third measurements, Figure 4A. Likewise, the Al potential initially stabilizes at approximately −0.18 V vs. SHE (−4.26 eV) before progressively approaching 0.00 V vs. SHE (−4.44 eV) in subsequent measurements. This evolution suggests gradual equilibration among the chemical potential of the electrode, sample, and SKP system (Figure 4A), with electrons flowing, from Al and ZnO@ITO toward the SKP through the Cu connection.

For the Cu/ZnO@FTO/Al configuration, the chemical potentials of Cu and ZnO@FTO were aligned throughout the sample, reaching a common value of approximately 0.35 V vs. SHE (−4.79 eV) in the first measurement and 0.27 V vs. SHE (−4.71 eV) in subsequent measurements (Figure 4C). Although the initial potential alignment across the sample suggests that the components approached electrochemical equilibrium at approximately 0.35 V vs. SHE (−4.79 eV), the strong electron-accepting character of ZnO promotes electron transfer from the FTO to the ZnO layer, driving the potential toward more negative values (Figure 4C). This process is further assisted by charge exchange between the sample and the SKP during the measurement. Consistent with this interpretation, the *nm*-SKPM measurements show that ZnO@FTO exhibits a potential of −0.32 V vs. SHE (−4.12 eV) (Figure 4D), which is more negative than both bare FTO and bare ZnO, providing independent evidence of charge redistribution within ZnO@FTO, as previously observed in the ZnO@ITO system (Figure 4B). A small peak of potential was observed at the Cu/ZnO@FTO interface (Figure 4C), although its amplitude was lower than that observed for ZnO@ITO (Figure 4A). In contrast, a pronounced potential wall appeared at the ZnO@FTO/Al interface (Figure 4C), accompanied by periodic oscillations extending across the Al region, as previously observed in the bare FTO, Figure S5C, Supplementary Information. The wavelengths of Al oscillations in Cu/ZnO@FTO/Al were similar across the measurements, ranging from approximately 975 μm in the first measurement to 1130 μm in the second and third measurements (Figure 4C).

In the case of the Cu/TiO_2_@ITO/Al configuration (Figure 4E), a similar tendency to that observed for the ZnO@ITO system is found (Figure 4A), with the chemical potentials of the TiO_2_@ITO layer and Cu aligning. The Cu chemical potential averages approximately 0.27 V vs. SHE (−4.71 eV), while the TiO_2_@ITO layer exhibits an average chemical potential of 0.30 V vs. SHE (−4.74 eV) (Figure 4E). At the nanoscale SKP, measurements show that the TiO_2_@ITO surface exhibits a potential of 0.22 vs. SHE (−4.66 eV) (Figure 4F). Unlike the ZnO@ITO system, the absence of a pronounced shift in the TiO_2_ surface potential indicates that no significant electron transfer occurs from the ITO to the TiO_2_ layer. Consequently, the measured surface potential remains close to the intrinsic chemical potential of TiO_2_ rather than exhibiting the negative shift associated with interfacial charge redistribution (Figure 4F). This interpretation is further supported by the agreement between the measured surface potential and values reported in the literature for TiO_2_ [39]. Additionally, as observed for the previous systems, charge accumulation regions are detected at both the Cu/TiO_2_@ITO and Al/TiO_2_@ITO interfaces, with the most pronounced potential peak occurring at the Al/TiO_2_@ITO interface (Figure 4E). Unlike ZnO, TiO_2_ exhibits a lower ability to conduct electrons across its surface. Consequently, TiO_2_ cannot efficiently channel the electrons originating from the Al substrate through its surface toward the remaining layers, resulting in enhanced charge accumulation at the Al/TiO_2_@ITO interface (Figure 4E). The ZnO@ITO/PET and TiO_2_@ITO/PET systems exhibit the same behavior, with ZnO showing a greater ability to conduct electrons across its surface than TiO_2_ (Figure S6A–D, Supplementary Information).

For the Cu/TiO_2_@FTO/Al configuration, the TiO_2_@FTO layer exhibited a potential of approximately 0.45 V vs. SHE (−4.89 eV) (Figure 4G), whereas the nanometric measurement holds a lower value of 0.20 V vs. SHE (−4.64 eV) (Figure 4H). Once again, the SKP measurement predominantly reflects the TiO_2_ surface, as this value corresponds to that expected for TiO_2_ alone [36]. Compared with the potential of bare FTO 0.33 V vs. SHE (−4.77 eV) (Figure S5D, Supplementary Information), the nanometric result 0.20 V vs. SHE (−4.64 eV) (Figure 4H) indicates only a small contribution from the underlying FTO and limited electron transfer to TiO_2_. Across successive SKP measurements, the chemical potentials of all components shifted downward, corresponding to increasingly positive potentials: from 0.10 to 0.21 V vs. SHE (−4.54 to −4.65 eV) for Cu, from 0.38 to 0.45 V vs. SHE (−4.82 to −4.89 eV) for TiO_2_@FTO, and from −0.12 to −0.01 V vs. SHE (−4.32 to −4.43 eV) for Al, Figure 4G. This coordinated shift is consistent with progressive electron transfer towards the Cu/SKP connection as the system becomes more positive and tries to align the potentials. Compared with ZnO, TiO_2_ appears less effective at accepting and retaining electrons from FTO, limiting negative surface charging and interfacial charge redistribution (Figure 4G). The more pronounced resistive character of the TiO_2_@FTO junction, shown in sheet resistance measurements (Figure 3G,J), may restrict lateral charge transport toward the other materials.

The optical response of the bare TCO substrates is presented in Figure S7A–C (Supplementary Information). PET/ITO, ITO and FTO glass exhibit high transparency throughout the visible region, with average transmittance values of approximately 84.2%, 83.2% and 79.4%, respectively, while maintaining low average reflectance (~10%) and absorptance below 11%. Within the UV region of the measured spectral range, all three substrates show a pronounced variation in absorptance and transmittance, consistent with the wide optical bandgaps reported for ITO and FTO, typically around 3.82–4.35 eV [5,7,8], which shift their fundamental absorption toward shorter wavelengths.

**Figure 5:**
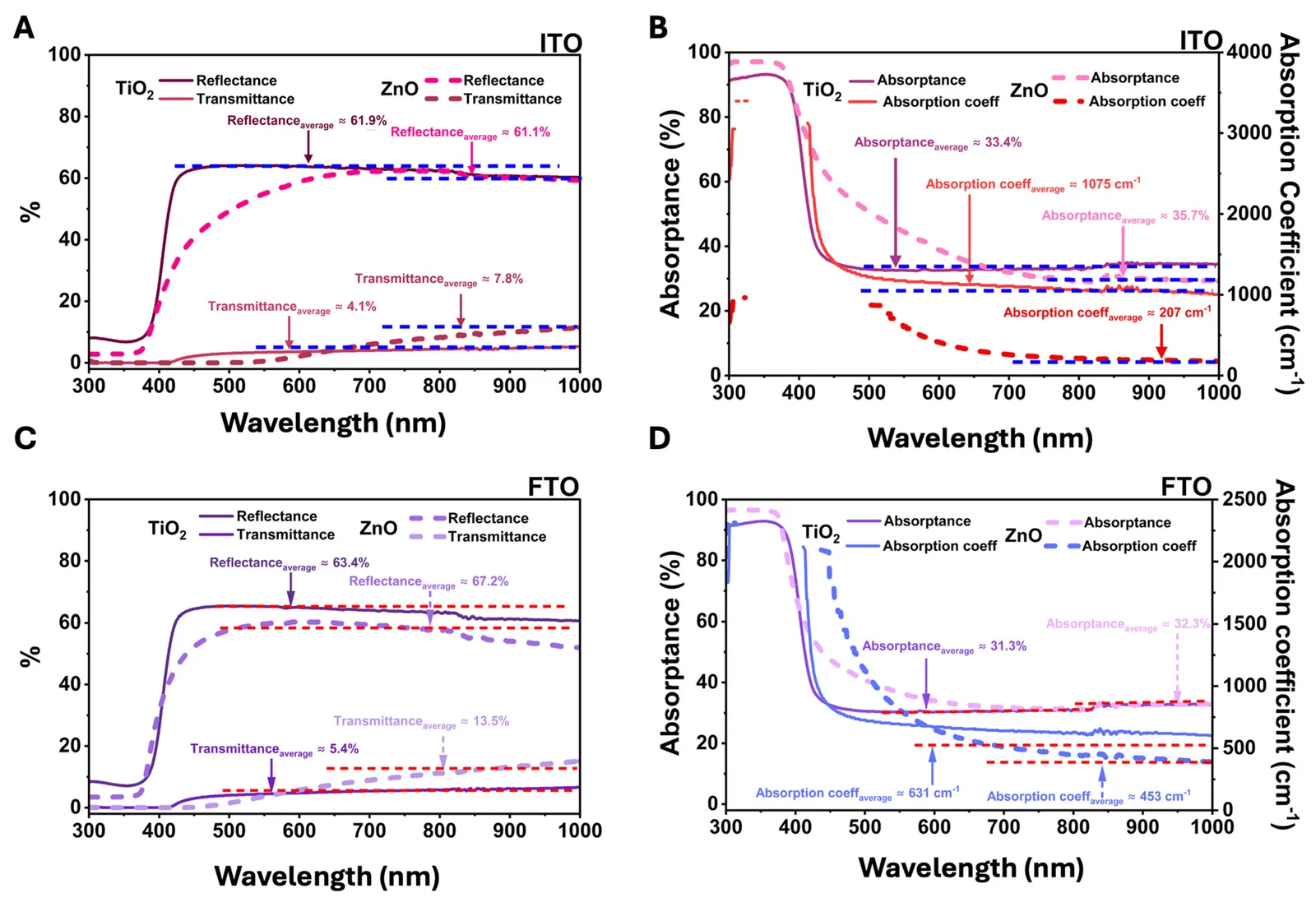
UV–visible spectroscopy (300–1000 nm) of ITO and FTO substrate coated with oxide layer. (**A**) Reflectance and transmittance of ZnO@ITO and TiO_2_@ITO. (**B**) Absorptance and absorption coefficient of ZnO@ITO and TiO_2_@ITO. (**C**) Reflectance and transmittance of ZnO@FTO and TiO_2_@FTO. (**D**) Absorptance and absorption coefficient of ZnO@FTO and TiO_2_@FTO. Average thicknesses: ZnO@ITO: 65 µm; ZnO@FTO: 34 µm; TiO_2_@ITO: 29 µm; TiO_2_@FTO: 28 µm. The absorptance was calculated via 1=T+R+A (left axis in Figure 5B,D), and the absorption coefficient was calculated using the following equation, $\alpha =\frac{1}{d}\ln\left(\frac{1-R}{T}\right)$, where d is thickness, R is reflectance and T is transmittance (right axis in Figure 5B,D).

Following the deposition of TiO_2_ and ZnO, the optical response changed considerably (Figure 5). All three substrates exhibited a significant reduction in transmittance and a simultaneous increase in reflectance (Figure 5) compared with their corresponding bare substrates, Figure S7A–C (Supplementary Information). The average reflectance increased to approximately 55–67% (Figure 5A,B and Figure S7D), whereas the transmittance decreased to values between 4.1 and 13.5% (Figure 5A,B and Figure S7D), depending on the deposited oxide and substrate. This behavior is expected considering the incorporation of relatively thick oxide layers of ~30–65 μm, as observed in Figure 2, Figures S1 and S2, Supplementary Information), which substantially modify the interaction of light with the TCO surface. The ZnO and TiO_2_ ETLs were fabricated by the drop coating deposition method, which, due to experimental limitations, did not allow for a significant reduction in layer thickness. Increasing the ETL thickness generally reduces optical transmittance, as widely reported in the literature. However, the objective of this work was not to optimize the thickness of the ETLs for photovoltaic device applications, but rather to investigate the interfacial interactions between the ETL materials and the TCO substrates. Consequently, the selected thicknesses were considered appropriate for the intended purpose of this study. The thickness values obtained from SEM and optical-microscope edge profiles are not identical because the drop-coated layers are spatially non-uniform and the two techniques probe different local regions and geometrical definitions of the coating edge. Accordingly, the reported values should be interpreted as representative local/average thicknesses rather than as a uniform film thickness over the entire sample.

Since the transmittance and reflectance were measured simultaneously and directly using a UV–Vis spectrometer, these data could not be normalized. However, the absorptance can be normalized to obtain the absorption coefficient, allowing a qualitative comparison of the influence of layer thickness, Figure 5B,D and Figure S7E, Supplementary Information. The absorption coefficient was calculated using the following equation, $\alpha =\frac{1}{d}\ln\left(\frac{1-R}{T}\right)$, where d is thickness, R is reflectance and T is transmittance (R and T were directly measured). It is important to note that, for all oxide/TCO combinations, the absorption coefficient cannot be calculated in the 300–400 nm wavelength range because the measured transmittance is zero. This behavior arises from the combined effect of the relatively large oxide layer thickness and the intrinsic bandgap of the oxide materials, which results in complete absorption of the incident light within the UV region. This behavior can be described by the following equation, $E=\frac{hc}{\lambda }$. On ITO, the average absorption coefficient was approximately 1075 cm^−1^ for TiO_2_ and 207 cm^−1^ for ZnO (Figure 5B), whereas on PET/ITO, the corresponding values were 304 and 228 cm^−1^, respectively (Figure S7E). In contrast, the absorption coefficients on FTO have a smaller gap between oxides, averaging 631 cm^−1^ for TiO_2_ and 453 cm^−1^ for ZnO (Figure 5D).

The use of absorptance and the derived absorption coefficient in this work is intended as a comparative treatment rather than a determination of intrinsic bulk optical constants. Because the drop-coated layers are rough, non-uniform, and optically scattering, the simple thickness normalization does not fully account for diffuse reflection, multiple scattering, porosity, or local variations in optical path length. Consequently, the calculated values should be interpreted as effective absorption coefficients that facilitate comparison among the deposited oxide/TCO systems. Here, “normalization” refers to accounting for the optical path length; the absorption coefficient is derived from the measured R and T values together with the coating thickness using the stated attenuation expression, rather than by direct division of absorptance by thickness.

On the three substrates, ZnO consistently exhibited higher average transmittance than TiO_2_—7.8% and 4.1% for ITO (Figure 5A), 13.5% and 5.4% for FTO (Figure 5C) and 11.2% and 4.7% for PET/ITO, respectively (Figure S7D)—while the average absorptance remained similar for both ZnO and TiO_2_ oxides—35.7 and 33.4% for ITO (Figure 5B), 32.3 and 31.3% for FTO (Figure 5D) and 28.3 and 30.5% for PET/ITO, respectively (Figure S7E). At shorter wavelengths, the coated systems exhibited high absorptance, which gradually decreased with increasing wavelength before stabilizing throughout the visible region (Figure 5). This behavior is consistent with the wide bandgaps of TiO_2_ and ZnO, commonly reported at approximately 3.0–3.8 eV [4,21,25,45].

The observed optical differences cannot be attributed solely to variations in coating thickness, as they remain evident in the thickness-normalized absorption coefficients, particularly for ITO and PET/ITO (Figure 5B,D and Figure S7E, Supplementary Information). The SEM images reveal clear differences in morphology, with ZnO forming a denser, more compact, robust layer, whereas TiO_2_ exhibits a rougher and less homogeneous surface (Figure 2 and Figure S2B–E). These morphological differences indicate that in addition to thickness, the microstructure of the deposited oxide layers plays an important role in determining the optical response of the ETL@TCO systems. Nevertheless, ZnO appears to be the most promising ETL for future photosensitive devices, as it transmits more light to the photoactive layer than TiO_2_. This is observed despite the ZnO layer being thicker and denser, while still exhibiting higher transmittance, lower absorptance and lower reflectance than TiO_2_.

**Figure 6:**
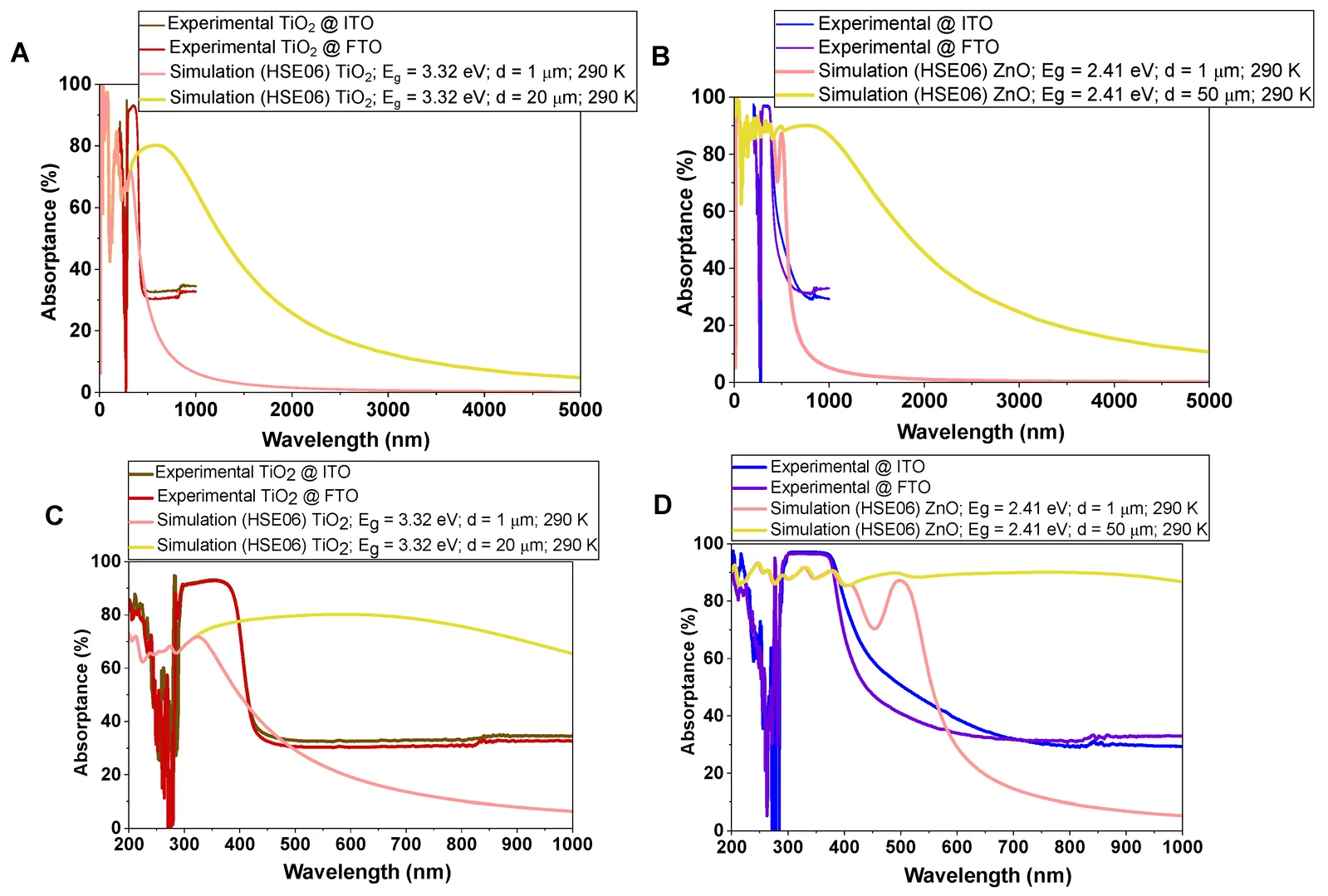
Experimental and simulated absorptance spectra of TiO_2_ and ZnO coatings on transparent conducting oxide substrates. (**A**) Experimental absorptance spectra of TiO_2_@ITO and TiO_2_@FTO compared with HSE06-based simulations for TiO_2_ with E_g_ = 3.32 eV, using layer thicknesses of 1 and 20 μm, over the 0–5000 nm wavelength range. (**B**) Experimental absorptance spectra measured on ITO and FTO substrates compared with HSE06-based simulations for ZnO with E_g_ = 2.41 eV, using thicknesses of 1 μm and 50 μm, over the 0–5000 nm wavelength range. (**C**,**D**) Magnified views of the ultraviolet–near-visible region between 200 and 1000 nm for the TiO_2_- and ZnO-based systems, respectively. The comparison highlights the strong experimental absorption features in the ultraviolet region and the thickness-dependent simulated absorption tails extending toward longer wavelengths.

Figure 6 compares the experimental absorptance spectra of the oxide-coated transparent conducting substrates with optical simulations based on the calculated HSE06 bandgaps. The comparison is useful because it separates two contributions that are experimentally superimposed: **(1)** the intrinsic optical response expected from the semiconductor bandgap and **(2)** the additional absorptance introduced by the conducting substrate, defects, thickness, scattering, and possible interfacial effects.

For the TiO_2_-based samples, the experimental spectra of TiO_2_@ITO and TiO_2_@FTO show strong absorptance in the ultraviolet region, followed by a rapid decrease toward the visible range. This behavior is consistent with the wide-band-gap nature of TiO_2_, for which the HSE06 calculation gives E_g_ = 3.32 eV. The corresponding absorption edge is expected in the near-ultraviolet, around 370–380 nm. The experimental spectra show intense features below approximately 400 nm, which agree with the onset of interband absorption in TiO_2_. However, the measured spectra do not follow the ideal simulated curves exactly. In particular, the experimental absorptance remains at a nearly constant level of about 30–35% at longer wavelengths. This residual absorptance is likely not due to the intrinsic TiO_2_ bandgap alone, but rather to contributions from the ITO/FTO substrates, free-carrier absorption, optical scattering, surface roughness, film thickness inhomogeneity, and possible defect or interfacial states.

The comparison between the 1 and 20 μm TiO_2_ simulations further illustrates the strong effect of optical path length. The thinner simulated layer shows a sharp decay after the band edge, whereas the thicker simulated layer produces a broad absorption tail extending into the near-infrared. The experimental spectra are much closer to the film response near the absorption edge but retain a finite long wavelength background. This indicates that the samples behave optically as absorbing oxide coatings on conducting transparent substrates rather than as ideal bulk TiO_2_ absorbers.

For the ZnO-based system, the HSE06 bandgap calculated in the simulations is E_g_ = 2.41 eV, corresponding to an absorption onset shifted to longer wavelengths compared with TiO_2_. The simulated ZnO spectra therefore show a broader response extending further into the visible region. The experimental spectra measured on ITO and FTO also show strong absorption and sharp spectral features in the ultraviolet–visible region, but again the agreement with the ideal simulations is only qualitative. In the 200–1000 nm region, the experimental curves display pronounced structure between approximately 250 and 400 nm, followed by a decrease toward a residual absorptance level. This suggests that the measured response is controlled not only by ZnO interband transitions but also by the transparent conducting substrate and by additional optical losses.

Standard HSE06 with the conventional 25% screened exact-exchange fraction is known to underestimate the bandgap of stoichiometric Wurtzite ZnO. Reported standard HSE06 values around 2.4–2.5 eV [49] are consistent with the present value of 2.41 eV, whereas reproducing the experimental optical gap near 3.2–3.4 eV generally requires a larger, material-specific exact-exchange fraction or a higher-level quasiparticle treatment. No defects, dopants, oxygen vacancies, or non-stoichiometric ZnO configurations were included here. The simulated spectrum is therefore interpreted as the internally consistent optical response of the standard HSE06 electronic structure, with emphasis on spectral shape and relative trends rather than exact reproduction of the experimental absorption onset.

The difference between ITO and FTO is also relevant. ITO is a degenerately doped n-type transparent conducting oxide, and its high free-electron concentration can introduce free-carrier absorption, especially toward longer wavelengths. FTO is also a doped transparent conducting oxide, but with a different carrier concentration, defect chemistry, and surface morphology. Therefore, even when the same oxide coating is considered, the substrate can modify the apparent absorptance through changes in reflectance, roughness, carrier absorption, and interface quality. The experimental differences between the ITO- and FTO-based samples should therefore be interpreted as a combined effect of the oxide layer and the conducting substrate.

The magnified spectra in Figure 6C,D emphasize that the ultraviolet–near-visible region is the most diagnostic part of the measurement. In this range, the experimental features coincide with the expected band-edge absorption of the oxide coatings. For TiO_2_, the absorption onset is consistent with a wide-gap semiconductor absorbing mainly in the ultraviolet. For ZnO, the lower simulated bandgap produces absorption extending further into the visible range. Nevertheless, the intensity and shape of the experimental curves deviate from the ideal HSE06-based simulations, confirming that real films contain additional optical contributions that are absent from the simplified model.

The results show that the calculated bandgaps correctly capture the expected spectral hierarchy: **(1)** TiO_2_ behaves as a wider-gap ultraviolet absorber, whereas **(2)** ZnO displays a lower-energy absorption onset. The simulations reproduce the main band-gap-controlled absorption trends, while the experimental spectra reveal the importance of film/substrate effects, defect states, thickness, scattering, and free-carrier absorption. Thus, the combined experimental/computational analysis supports the assignment of the dominant optical transitions while highlighting that transparent conducting oxide substrates cannot be treated as optically passive supports.

## 3. Materials and Methods

### 3.1. Materials

We have used the following semiconductor materials:

**TiO_2_** Titanium (IV) oxide rutile 99.9% metal basis, 3–6 mm sintered pieces ball milled to ≤44 μm, Thermo Scientific, Waltham, MA, USA.

**ZnO** Zinc oxide 99.9% metal basis powder, Alfa Aesar, Heysham, UK.

As substrate, the following materials were implemented:

**PET/ITO** commercial substrate consisting of 125 μm of thick PET substrate coated with a 0.200 µm thick ITO layer.

**FTO-coated glass substrate** commercial 25 × 25 mm with 1.1 mm of thickness and sheet resistance 15 Ohm/sq, MSE Suppliers, Tucson, AZ, USA. FTO thickness: 0.320–0.340 µm.

**ITO-coated glass substrate** commercial 25 × 25 mm with 1.1 mm of thickness and sheet resistance < 10 Ohm/sq, MSE Suppliers, Tucson, AZ, USA. ITO thickness: 0.120–0.160 µm.

### 3.2. Characterization Techniques

#### 3.2.1. Micrometer-Resolution SKP

Micrometer-resolution SKP was performed on samples with Cu/sample/Al configuration, to determine the surface chemical potential of the samples. μm-SKP measurements were conducted using an SKP-M470 (Biologic, Seyssinet-Pariset, France) system equipped with U-SKP-370/1 tips. Measurements were performed inside a controlled dry box environment, maintaining a tip-to-sample distance of 100 μm for topographical measurements and surface potential maps. The standard deviation for the μm-SKP: ± (0.02–0.16) eV [50].

#### 3.2.2. Nanometer-Resolution SKP

Nanometer-resolution SKP was performed on an MFP-3D Origin^+^ atomic force microscope (Oxford Instruments Asylum Research, Santa Barbara, CA, USA). This analysis employed a laser-driven ASYELEC-01-R2 tip, with a resonant frequency of ≈75 kHz. The tip was composed of silicon with a reflective Ti/Ir coating and had a spring constant of 2.8 N·m^−1^. Topography measurements were conducted in tapping mode, in which the tip gently oscillates over the sample surface to acquire high-resolution surface data. Simultaneously, nm-SKP data were collected to map the surface potential distribution. The measurements were performed on the ETL-coated TCOs sample, allowing direct comparison with the results obtained in μm-SKP.

The standard deviation for the *nm*-SKP: ± (0.050–0.100) eV [51].

#### 3.2.3. Sheet Resistance Analysis and Hall Effect Analysis

Hall effect measurements were carried out using an HCS 1 (Linseis Messgeräte GmbH, Selb, Germany) system equipped with two movable neodymium magnetic circuits, enabling the application of a perpendicular magnetic field of ±0.7 T. Standard deviation of the equipment: temperature ±0.05 °C; voltage ±20 nV, current 16 µA [52]. Electrical contacts were established at the edges of the samples, and a constant current in the range of 2 mA was applied. The Hall voltage ($V_{H})$, arising perpendicular to both the applied current and magnetic field, was recorded with high sensitivity. The Hall coefficient was calculated through $R_{H}=\frac{V_{H}}{IB}$, with B being the perpendicular magnetic field. Charge carrier density and mobility were respectively calculated as $n=\frac{1}{eR_{H}}$ and $\mu^{\prime }=\frac{R_{H}\sigma }{e}$, where e is the elementary charge and σ is the conductivity. Sheet resistance was evaluated using the van der Pauw method on samples with uniform thickness. Four collinear contacts were employed, and measurements were performed across multiple contact configurations to improve accuracy. The sheet resistance, $R_{s}=\frac{V}{I}f$, was calculated from the measured current and voltage using a geometrical correction factor (f), together with the van der Pauw relation $e^{-\pi Rs_{1}/Rs}+\;e^{-\pi Rs_{2}/Rs}=1$. All measurements were conducted under controlled conditions, with the chamber evacuated to ~10^−2^ bar and continuously purged with nitrogen gas (4–5 L·min^−1^) to minimize noise, temperature fluctuations, and undesired reactions. The measurements were performed on the ETL-coated TCO sample, with the four gold tips in direct contact with the ETL.

#### 3.2.4. UV–Visible Spectroscopy

UV–visible spectroscopy measurements were performed to evaluate the optical response of the samples. Spectra were acquired using an UV-3600i Plus spectrophotometer (Shimadzu Corporation, Kyoto, Japan) over a wavelength range from 200 to 1000 nm. Reflectance (R) and transmittance (T) measurements were conducted with a data interval of 1 nm at medium scan speed, using an automatic light source and detector switching system to ensure accurate signal acquisition across the full spectral range. The instrument was equipped with a multi-detector configuration (photomultiplier, InGaAs, and PbS detectors), enabling high sensitivity across the UV–visible–near-infrared regions. All measurements were carried out under standard conditions. Absorptance (A) spectrum was obtained through 1=A+T+R. The standard deviation of the spectrometer is ±0.2 nm [53].

#### 3.2.5. SEM/EDX

Morphological and compositional characterization was performed using scanning electron microscopy (SEM) coupled with energy-dispersive X-ray spectroscopy (EDX). SEM imaging was carried out using a QUANTA 400 FEG ESEM (FEI Company, Hillsboro, OR, USA), while elemental analysis was conducted with a Genesis X4M EDX system (EDAX Inc., Mahwah, NJ, USA). Microstructural observations were obtained under an accelerating voltage of 15 kV, with a spot size ranging from 3 to 5 and a working distance of approximately 10 μm. Images were acquired at magnifications of 500× for ZnO@TCO and 1200× for TiO_2_@TCO using a secondary electron detector (Everhart-Thornley Detector) (FEI Company, Hillsboro, OR, USA), enabling contrast between different phases and compositional variations within the samples.

#### 3.2.6. Digital Microscope

Surface morphology and film thickness were additionally examined using an Olympus DSX2000 digital microscope (Evident Corporation, Tokyo, Japan) operated in bright-field mode with a 20× objective. Three-dimensional surface profiles were acquired to determine the deposition height (film thickness), while two-dimensional surface profiles were obtained to assess surface morphology and uniformity across the samples.

### 3.3. Computational Details

First-principles calculations were performed to compare the electronic structures, surface energetics, and optical responses of indium tin oxide (ITO), fluorine-doped tin oxide (FTO), ZnO and TiO_2_. The ITO and FTO models were represented by the doped supercells In_29_Sn_3_O_48_ and Sn_16_O_29_F_3_, which account explicitly for substitutional Sn^4+^ on In^3+^ sites in ITO and F^−^ on O^2−^ sites in FTO. These substitutions introduce excess electrons and reproduce the degenerately doped n-type character of the transparent conducting oxides observed experimentally.

Periodic DFT and hybrid HSE06 simulations were performed using the projector-augmented-wave method, as implemented in Vienna Ab initio Simulation Package (VASP 6). Structural relaxations were obtained using the PBE-GGA exchange–correlation functional with electronic iterations convergence of 10^−5^ eV and k-spacing for non-local exchange 0.2–0.3 Å^−1^. A plane-wave cutoff energy of 400–520 eV and Monkhorst–Pack scheme were used. The same convergence criteria were applied consistently to the four oxide systems.

#### 3.3.1. Electronic Density of States

The electronic density of states (DOS) was calculated for the relaxed ITO and FTO structures and normalized by the number of atoms in each simulation cell. The total and element-resolved DOSs were aligned relative to the Fermi level to identify the valence band region, conduction band states, and dopant-derived donor levels. Particular attention was given to the low-density region close to the conduction band minimum, where Sn-derived states in ITO and F-derived states in FTO contribute to the finite DOS around the Fermi level and to the degenerate n-type behavior.

Because semilocal density functional approximations tend to underestimate oxide bandgaps, the calculated valence to conduction band separations were interpreted mainly in terms of comparative electronic structure and donor-state distribution. The simulations yielded separations of approximately 2.4 eV for ITO and 2.2 eV for FTO, while preserving the expected doped-conductor character of both materials. To avoid the latter underestimation, periodic structures of TiO_2_ and ZnO and their electronic and optical properties were simulated using HSE06.

#### 3.3.2. Surface Models and Work Functions

Surface slabs were built for the principal low-index orientations of ITO and FTO. For ITO, the (001)/(100), (110), and (111) surfaces were considered, including a termination in which a Sn^4+^ dopant was located near the surface. For FTO, the (100)/(010), (001), and (110) surfaces were investigated, together with a termination containing a surface-near F^−^ dopant. A vacuum region of 10 Å was introduced normal to the surface to avoid interactions between periodic images.

The planar-averaged electrostatic potential, $<V>$, was calculated along the surface-normal coordinate z,

(1) $$<V>=\frac{1}{A}\int_{A}V\left(x,y,z\right)dxdy$$

where A is the surface area of the simulation cell. A macroscopic average was subsequently applied to suppress atomic-scale oscillations and identify the asymptotic vacuum level, $E_{vac}$ = 0 eV. The work function WF was determined from

(2) $$WF=\Phi =E_{vac}-E_{F}$$

where $E_{F}$ is the Fermi level. The corresponding electronic chemical potential, μ, on the absolute vacuum scale was taken as

(3) μ=−Φ

The most representative calculated work functions were approximately 4.7 eV for ITO and 5.9 eV for FTO, although variations were obtained among facets and dopant terminations because of differences in surface coordination and surface dipoles. These values indicate a deeper Fermi level for FTO and therefore different charge-transfer driving forces when the conducting oxides are placed in contact with ZnO or TiO_2_.

Where necessary, vacuum-referenced chemical potentials were converted to the standard hydrogen electrode scale, $E_{SHE}$, according to

(4) $$E_{SHE}=-\left(\mu +4.44\right)\;V$$

using 4.44 eV as the absolute energy of the standard hydrogen electrode.

#### 3.3.3. Hybrid-Functional Band Structures and Optical Response

The intrinsic electronic and optical responses of ZnO and TiO_2_ were evaluated using the screened hybrid HSE06 functional to improve the description of the bandgaps relative to semilocal DFT. The calculations yielded bandgaps of approximately 2.41 eV for ZnO and 3.32 eV for TiO_2_. These values were used to establish the relative positions of the fundamental optical absorption edges and to simulate the idealized spectral response of the oxide layers.

For ZnO, the relaxed stoichiometric Wurtzite structure with space group P63mc was used. The Zn and O PAW datasets, plane-wave cutoff, reciprocal-space sampling, and electronic convergence were examined, and the 2.41 eV gap was found to be numerically stable within the adopted standard HSE06 setup. The lower value relative to experiment is therefore attributed to the functional rather than to a different crystal phase, defect model, or misidentified transition.

The frequency-dependent complex dielectric function, $\varepsilon \left(\omega \right)$, was expressed as

(5) $$\varepsilon \left(\omega \right)=\varepsilon_{1}\left(\omega \right)+i\varepsilon_{2}\left(\omega \right)$$

where $\varepsilon_{1}\left(\omega \right)$ is the real and $\varepsilon_{2}\left(\omega \right)$ imaginary permittivities, respectively, from which the complex refractive index, $\tilde{n}\left(\omega \right)$, was obtained.

(6) $$\tilde{n}\left(\omega \right)=n_{1}\left(\omega \right)+in_{2}\left(\omega \right)$$

where $n_{1}\left(\omega \right)$ and $n_{2}\left(\omega \right)$ are the real and imaginary parts of the complex refractive index, respectively. The extinction coefficient $k\left(\omega \right)$ was calculated from

(7) $$k\left(\omega \right)={\left(\frac{\sqrt{\varepsilon_{1}^{2}+\varepsilon_{2}^{2}}-\varepsilon_{1}}{2}\right)}^{1/2}$$

and the absorption coefficient $\alpha \left(\omega \right)$ was determined according to

(8) $$\alpha \left(\omega \right)=\frac{2\omega k\left(\omega \right)}{c}=\frac{4\pi k\left(\lambda \right)}{\lambda }$$

where *c* is the velocity of the light and λ the wavelength.

Simulated spectra were generated for TiO_2_ layer thicknesses of 1 and 20 µm and for ZnO layer thicknesses of 1 and 50 µm. The spectra were calculated over the 0–5000 nm wavelength interval, with particular attention to the 200–1000 nm region used in the experimental UV–visible measurements. The calculated curves were compared qualitatively with the absorptance spectra measured for TiO_2_@ITO, TiO_2_@FTO, ZnO@ITO and ZnO@FTO.

The simulations were not intended to reproduce the absolute experimental absorptance of the complete ETL/TCO assemblies. Instead, they provide a reference for separating the intrinsic absorption associated with the ZnO and TiO_2_ electronic band structures from extrinsic contributions arising from the ITO or FTO substrate, free carriers, surface defects, interfacial states, film thickness variations, morphology, and optical scattering.

The optical simulations were derived from the frequency-dependent complex dielectric function and the corresponding refractive index, extinction coefficient, and absorption coefficient. The Beer–Lambert attenuation relation was subsequently used only to estimate thickness-dependent idealized absorptance. Therefore, the calculated curves do not include the roughness, porosity, diffuse scattering, substrate absorption, free-carrier response, or interface-specific optical losses present in the experimental assemblies.

## 4. Conclusions

This work demonstrates that ITO and FTO are not passive supports in ZnO- and TiO_2_-based heterojunctions. Although both are degenerately doped n-type transparent conducting oxides, their different work functions, surface dipoles, and donor-state distributions produce distinct interfacial charge-transfer behavior. ZnO promotes stronger electron accumulation and more pronounced temperature-dependent transport transitions, whereas TiO_2_ exhibits weaker electron uptake and a more resistive response. FTO-based heterojunctions display clearer thermal hysteresis, consistent with the greater thermal stability of FTO. Optical measurements further show that ZnO provides higher visible transmittance than TiO_2_, while the deviations from ideal HSE06-based spectra shape and relative trends reflect the effects of substrate absorption, defects, roughness, thickness, scattering, and free carriers. The results show that the performance of TCO/ETL interfaces is governed by the coupled electronic, thermal, and optical response of the substrate, oxide layer, and interface, providing guidance for the design of transparent electrodes and optoelectronic heterojunctions.

## Data Availability

Data available upon request.

## Supplementary Materials

The following supporting information can be downloaded at: https://www.mdpi.com/article/10.3390/molecules31162868/s1. Table S1. Energy–dispersive X–ray spectroscopy (EDX) analysis of the deposited oxide layers in Figure 2. Figure S1. Morphological characterization of bare and oxide-coated ITO and FTO substrates. Scanning electron microscopy images of bare transparent conducting oxide substrates: (A) ITO and (B) FTO. Optical microscope cross-sectional images of deposited oxide layers on ITO and FTO: (C) ZnO@ITO, (D) ZnO@FTO, (E) TiO_2_@ITO, and (F) TiO_2_@FTO. The images show the surface morphology of the bare substrates and the approximate thickness/edge profile of the ZnO and TiO_2_ coatings deposited on each transparent electrode. Note: These are local approximate thicknesses obtained at different positions and by different geometrical methods on spatially non-uniform drop-coated layers, rather than values expected to coincide exactly with the SEM averages. Figure S2. Morphological characterization of bare and oxide-coated PET/ITO substrates. (A) SEM image of the bare PET/ITO substrate. (B,C) Optical microscope image and corresponding three-dimensional surface reconstruction of the ZnO layer deposited on PET/ITO. (D,E) Optical microscope image and corresponding three-dimensional surface reconstruction of the TiO_2_ layer deposited on PET/ITO. The measurements provide the approximate coating thickness and surface profile of the oxide layers on the flexible transparent conducting substrate. Figure S3. Temperature-dependent electrical properties of bare and oxide-coated transparent conducting substrates. Sheet resistance, charge carrier concentration, and carrier mobility measured during heating and cooling for (A–C) bare ITO, (D–F) FTO, (G–I) PET/ITO, and (J–O) oxide-coated PET/ITO systems. Panels include the electrical response of the bare transparent conducting oxides and the corresponding TiO_2_- and ZnO-coated heterojunctions, allowing comparison of substrate-dominated and oxide- interface-dominated transport regimes. Figure S4. Thermal hysteresis in the sheet resistance of TiO_2_- and ZnO-coated transparent conducting substrates. Temperature-dependent sheet resistance during heating and cooling for (A) TiO_2_@ITO, (B) ZnO@ITO, (C) TiO_2_@FTO, (D) ZnO@FTO, (E) TiO_2_@PET/ITO, and (F) ZnO@PET/ITO. The shaded regions highlight the hysteretic behavior associated with thermally induced changes in the transport response of the oxide-coated heterojunctions. Figure S5. Surface-potential characterization of bare ITO, FTO, and PET/ITO substrates. Micrometric-resolution scanning Kelvin probe profiles and nanometric-resolution surface-potential maps of bare transparent conducting substrates. (A,B) ITO, (C,D) FTO, and (E,F) PET/ITO. The micrometric profiles were obtained in Cu/TCO/Al configurations, while the nanometric maps were acquired directly on the TCO surfaces, providing reference chemical-potential values for comparison with oxide-coated systems. Figure S6. Surface-potential characterization of oxide-coated PET/ITO substrates. Micrometric-resolution scanning Kelvin probe profiles and nanometric-resolution surface-potential maps of oxide-coated PET/ITO heterojunctions. (A,B) TiO_2_@PET/ITO and (C,D) ZnO@PET/ITO. The measurements compare the interfacial charge redistribution induced by TiO_2_ and ZnO coatings on the flexible PET/ITO transparent electrode. Figure S7. Optical response of bare and oxide-coated transparent conducting substrates. UV–visible reflectance, transmittance, and absorptance spectra of bare (A) ITO, (B) FTO, and (C) PET/ITO substrates. (D) Reflectance and transmittance spectra of TiO_2_@PET/ITO and ZnO@PET/ITO. (E) Absorptance and absorption coefficient spectra of TiO_2_@PET/ITO and ZnO@PET/ITO. These data complement the optical analysis of the glass-based ITO and FTO substrates by showing the behavior of the flexible PET/ITO platform. Note: Average thicknesses: PET/ITO; ITO thickness: 0.200 µm thick ITO layer; ZnO@PET/ITO: 61 µm; TiO_2_@PET/ITO: 58 µm.

## References

[1] Vega-Garita V., Ramirez-Elizondo L., Narayan N., Bauer P. (2018). Integrating a Photovoltaic Storage System in One Device: A Critical Review. Prog. Photovolt. Res. Appl..

[2] Tian R., Zhou S., Meng Y., Liu C., Ge Z. (2024). Material and Device Design of Flexible Perovskite Solar Cells for Next–Generation Power Supplies. Adv. Mater..

[3] Cheng M., Zuo C., Wu Y., Li Z., Xu B., Hua Y., Ding L. (2020). Charge–Transport Layer Engineering in Perovskite Solar Cells. Sci. Bull..

[4] Sharma H., Srivastava R. (2023). Solution–Processed Pristine Metal Oxides as Electron–Transporting Materials for Perovskite Solar Cells. Front. Electron. Mater..

[5] Zhang B., Dong X., Xu X., Wang X., Wu J. (2007). Electrical and Optical Properties of ITO and ITO:Zr Transparent Conducting Films. Mater. Sci. Semicond. Process..

[6] Alam M., Cameron D. (2000). Optical and Electrical Properties of Transparent Conductive ITO Thin Films Deposited by Sol–Gel Process. Thin Solid Film..

[7] Gonçalves G., Elamurugu E., Barquinha P., Pereira L., Martins R., Fortunato E. (2007). Influence of Post–Annealing Temperature on the Properties Exhibited by ITO IZO and GZO Thin Films. Thin Solid Films.

[8] Kahattha C., Noonuruk R., Pecharapa W. (2016). Influence of Annealing Temperature on Optical Properties of Fluoride Doped Tin Oxide Films Grown by the Sol–Gel Spin–Coating Method. Integr. Ferroelectr..

[9] Sofi A.H., Shah M.A., Asokan K. (2018). Structural, Optical and Electrical Properties of ITO Thin Films. J. Electron. Mater..

[10] Ali A.H., Hassan Z., Shuhaimi A. (2018). Enhancement of Optical Transmittance and Electrical Resistivity of Post–Annealed ITO Thin Films RF Sputtered on Si. Appl. Surf. Sci..

[11] Złotnik S., Pianelli A., Boguski J., Kojdecki M.A., Moszczyński P., Parka J., Wróbel J. (2022). Experimental Verification of Single–Type Electron Population in Indium Tin Oxide Layers. Phys. Status Solidi (RRL)–Rapid Res. Lett..

[12] Damgaci E., Kartal E., Gucluer F., Seyhan A., Kaplan Y. (2024). Impact of Temperature Optimization of ITO Thin Film on Tandem Solar Cell Efficiency. Materials.

[13] Nishimoto N., Yamada Y., Ohnishi Y., Imawaka N., Yoshino K. (2013). Effect of Temperature on the Electrical Properties of ITO in a TiO_2_/ITO Film. Phys. Status Solidi.

[14] Xu Z., Xue T., Guo Q., Yao J., Li G., Du J., Zhou E. (2025). Emerging Flexible Photovoltaic Technology: From Materials to Devices. Inf. Funct. Mater..

[15] Yang D., Yang R., Priya S., Liu S. (2019). Recent Advances in Flexible Perovskite Solar Cells: Fabrication and Applications. Angew. Chem. Int. Ed..

[16] Vinutha K.V., Naveen Kumar K.B., Tejas M.K., Kai Kumar B., Sumanth Kumar D., Mahesh M.H. (2016). Natural Dye Sensitized Solar Cells Using Anthocyanin Pigment of Strawberry as Sensitizers. Imp. J. Interdiscip. Res..

[17] Wang J.T., Shi X.L., Liu W.W., Zhong X.H., Wang J.N., Pyrah L., Sanderson K.D., Ramsey P.M., Hirata M., Tsuri K. (2014). Influence of Preferred Orientation on the Electrical Conductivity of Fluorine–Doped Tin Oxide Films. Sci. Rep..

[18] Yang J.K., Liang B., Zhao M.J., Gao Y., Zhang F.C., Zhao H.L. (2015). Reference of Temperature and Time during Tempering Process for Non–Stoichiometric FTO Films. Sci. Rep..

[19] Jafari S., Mahyad B., Hashemzadeh H., Janfaza S., Gholikhani T., Tayebi L. (2020). Biomedical Applications of TiO_2_ Nanostructures: Recent Advances. Int. J. Nanomed..

[20] Morsella M., d’Alessandro N., Lanterna A., Scaiano J. (2016). Improving the Sunscreen Properties of TiO_2_ through an Understanding of Its Catalytic Properties. ACS Omega.

[21] Li Z., Li Z., Zuo C., Fang X. (2022). Application of Nanostructured TiO_2_ in UV Photodetectors: A Review. Adv. Mater..

[22] Fujishima A., Honda K. (1972). Electrochemical Photolysis of Water at a Semiconductor Electrode. Nature.

[23] Nair R., Gummaluri V.S., Vadakke Matham M., Vijayan C. (2022). A Review on Optical Bandgap Engineering in TiO_2_ Nanostructures via Doping and Intrinsic Vacancy Modulation towards Visible Light Applications. J. Phys. D Appl. Phys..

[24] Zhao W., Guo P., Wu J., Lin D., Jia N., Fang Z., Liu C., Ye Q., Zou J., Zhou Y. (2024). TiO_2_ Electron Transport Layer with P–n Homojunctions for Efficient and Stable Perovskite Solar Cells. Nano-Micro Lett..

[25] Qiu C., Wu Y., Song J., Wang W., Li Z. (2022). Efficient Planar Perovskite Solar Cells with ZnO Electron Transport Layer. Coatings.

[26] Kumar A., Kumar D., Jain N., Kumar M., Ghodake G., Kumar S., Sharma R.K., Holovsky J., Saji V.S., Sharma S.K. (2023). Enhanced Efficiency and Stability of Electron Transport Layer in Perovskite Tandem Solar Cells: Challenges and Future Perspectives. Sol. Energy.

[27] Drygała A., Starowicz Z., Gawlińska-Nęcek K., Karolus M., Lipiński M., Jarka P., Matysiak W., Tillová E., Palček P., Tański T. (2023). Hybrid Mesoporous TiO_2_/ZnO Electron Transport Layer for Efficient Perovskite Solar Cell. Molecules.

[28] Adnan M., Usman M., Ali S., Javed S., Islam M., Akram M.A. (2022). Aluminum Doping Effects on Interface Depletion Width of Low Temperature Processed ZnO Electron Transport Layer–Based Perovskite Solar Cells. Front. Chem..

[29] Nath B., Uppara B., Singh S., Ramamurthy P.C., Roy Mahapatra D., Hegde G. (2024). Exploring the Potential of Green Synthesized ZnO–SnO_2_ Composite as an Effective Electron Transport Layer for Perovskite Solar Cells: A Sustainable Approach. Chem. Eng. Sci..

[30] Jarwal D.K., Mishra A.K., Baral K., Kumar A., Kumar C., Rawat G., Mukherjee B., Jit S. (2022). Performance Optimization of ZnO Nanorods ETL Based Hybrid Perovskite Solar Cells with Different Seed Layers. IEEE Trans. Electron. Devices.

[31] Guerreiro A.N., Baptista M.C., Maia B.A., Braga M.H. (2022). Interfacial Chemistry with ZnO: In Operando Work Functions in Heterocells. ACS Appl. Energy Mater..

[32] Liu H., Zhu Y., Xiong W., Yang Y., Li S., Bai S. (2025). Transparent Conductive Oxide Films and Their Applications in Silicon Heterojunction and Perovskite/Silicon Tandem Photovoltaics. ACS Appl. Energy Mater..

[33] Yao H., Pugliese D., Lancry M., Dai Y. (2024). Ultrafast Laser Direct Writing Nanogratings and their Engineering in Transparent Materials. Laser Photon-Rev..

[34] Yao H., Xie Q., Cavillon M., Dai Y., Lancry M. (2023). Materials roadmap for inscription of nanogratings inside transparent dielectrics using ultrafast lasers. Prog. Mater. Sci..

[35] Yao H., Ari J., Pugliese D., Dussauze M., Qiu J., Lancry M. (2026). Ultrafast laser-induced refractive index tuning inside GeSbSNa glasses for mid-IR applications. Opt. Express.

[36] Klein A., Körber C., Wachau A., Säuberlich F., Gassenbauer Y., Harvey S.P., Proffit D.E., Mason T.O. (2010). Transparent Conducting Oxides for Photovoltaics: Manipulation of Fermi Level, Work Function and Energy Band Alignment. Materials.

[37] Li C., Zhang Y., Bi W., Ren Z., Han G., Wang L., Ma S. (2026). One–Step Thermal Engineering of FTO Substrate: Unlocking Higher–Efficiency Perovskite Solar Cells. Appl. Energy.

[38] Seok H.-J., Ali A., Seo J.H., Lee H.H., Jung N.-E., Yi Y., Kim H.-K. (2019). ZnO:Ga–Graded ITO Electrodes to Control Interface between PCBM and ITO in Planar Perovskite Solar Cells. Sci. Technol. Adv. Mater..

[39] Gomes B.M., Holtz J., Pinto M.L., Braga M.H. (2026). Polaronic and Electrochemical Signatures in Group IVB (Ti, Zr, Hf) Oxides: Unified SKP–DFT Insights for Tunable Transport in Energy and Electronic Devices. Adv. Funct. Mater..

[40] Park Y., Choong V., Gao Y., Hsieh B.R., Tang C.W. (1996). Work Function of Indium Tin Oxide Transparent Conductor Measured by Photoelectron Spectroscopy. Appl. Phys. Lett..

[41] Zhou Y., Shim J.W., Fuentes–Hernandez C., Sharma A., Knauer K.A., Giordano A.J., Marder S.R., Kippelen B. (2012). Direct Correlation between Work Function of Indium–Tin–Oxide Electrodes and Solar Cell Performance Influenced by Ultraviolet Irradiation and Air Exposure. Phys. Chem. Chem. Phys..

[42] Nehate S.D., Prakash A., Mani P.D., Sundaram K.B. (2018). Work Function Extraction of Indium Tin Oxide Films from MOSFET Devices. ECS J. Solid State Sci. Technol..

[43] Helander M., Greiner M., Wang Z.B., Tang W., Lu Z.H. (2011). Work Function of Fluorine Doped Tin Oxide. J. Vac. Sci. Technol. A Vac. Surf. Film..

[44] Kashiwaya S., Morasch J., Streibel V., Toupance T., Jaegermann W., Klein A. (2018). The Work Function of TiO_2_. Surfaces.

[45] Kavan L. (2024). Electrochemistry and Band Structure of Semiconductors (TiO_2_, SnO_2_, ZnO): Avoiding Pitfalls and Textbook Errors. J. Solid State Electrochem..

[46] Musa I. (2024). Kelvin Probe Force Microscopy, Current Mapping, and Optical Properties of Hybrid ZnO Nanorods/Ag Nanoparticles. Surfaces.

[47] Krysova H., Mansfeldova V., Tarabkova H., Pisarikova A., Hubicka Z., Kavan L. (2024). High–Quality Dense ZnO Thin Films: Work Function and Photo/Electrochemical Properties. J. Solid State Electrochem..

[48] Guerreiro A.N., Costa I.B., Vale A.B., Braga M.H. (2023). Distinctive Electric Properties of Group 14 Oxides: SiO_2_, SiO, and SnO_2_. Int. J. Mol. Sci..

[49] Oba F., Choi M., Togo A., Tanaka I. (2011). Point defects in ZnO: An approach from first principles. Sci. Technol. Adv. Mater..

[50] Perform Scanning Kelvin Probe Measurements on BioLogic’s Modular M470 with the SKP470 Option. https://www.biologic.net/products/skp-m470/.

[51] MFP-3D Origin+ AFM. https://afm.oxinst.com/products/mfp-3d-afm-systems/mfp-3d-origin-plus-afm.

[52] Characterization of Semiconductors and Devices. https://shop.linseis.com/Measuring-instruments-Basic-line/Hall-Effect-Analyzer/HCS-1/.

[53] UV-3600i Plus Spectrophotometer. https://www.ssi.shimadzu.com/products/uv-vis/uv-vis-nir-spectroscopy/uv-3600i-plus/index.html.

