# Supplementary material for: From Transparency to Transport: Optoelectronic and Interfacial Signatures of n-Type ITO, FTO, ZnO and TiO2 Semiconductors
Source: Molecules. 2026 Aug 17;31(16):2868. doi: 10.3390/molecules31162868 (PMC13515800; doi:10.3390/molecules31162868)
Supplement: Supplementary file 1 [file molecules-31-02868-s001.zip › molecules-4480952-supplementary.pdf]

Supplementary Information

From Transparency to Transport: Optoelectronic and Interfacial Signatures of n-Type ITO, FTO, ZnO and TiO_2 Semiconductors

Júlia Holtz, Beatriz Moura Gomes, Vera C. M. Duarte, Joana Figueira, Joana Vaz Pinto, Luísa Andrade, Maria Helena Braga

Table S1. Energy−dispersive X−ray spectroscopy (EDX) analysis of the deposited oxide layers in Figure 2.

Zones	At %	
	Zn	O
**Figure 2A - Zone1**	72.15%	27.85%
**Figure 2B - Zone2**	71.70%	28.30%

Zones	At %	
	Ti	O
**Figure 2C - Zone3**	43.85%	56.15%
**Figure 2D - Zone4**	43.54%	56.46%

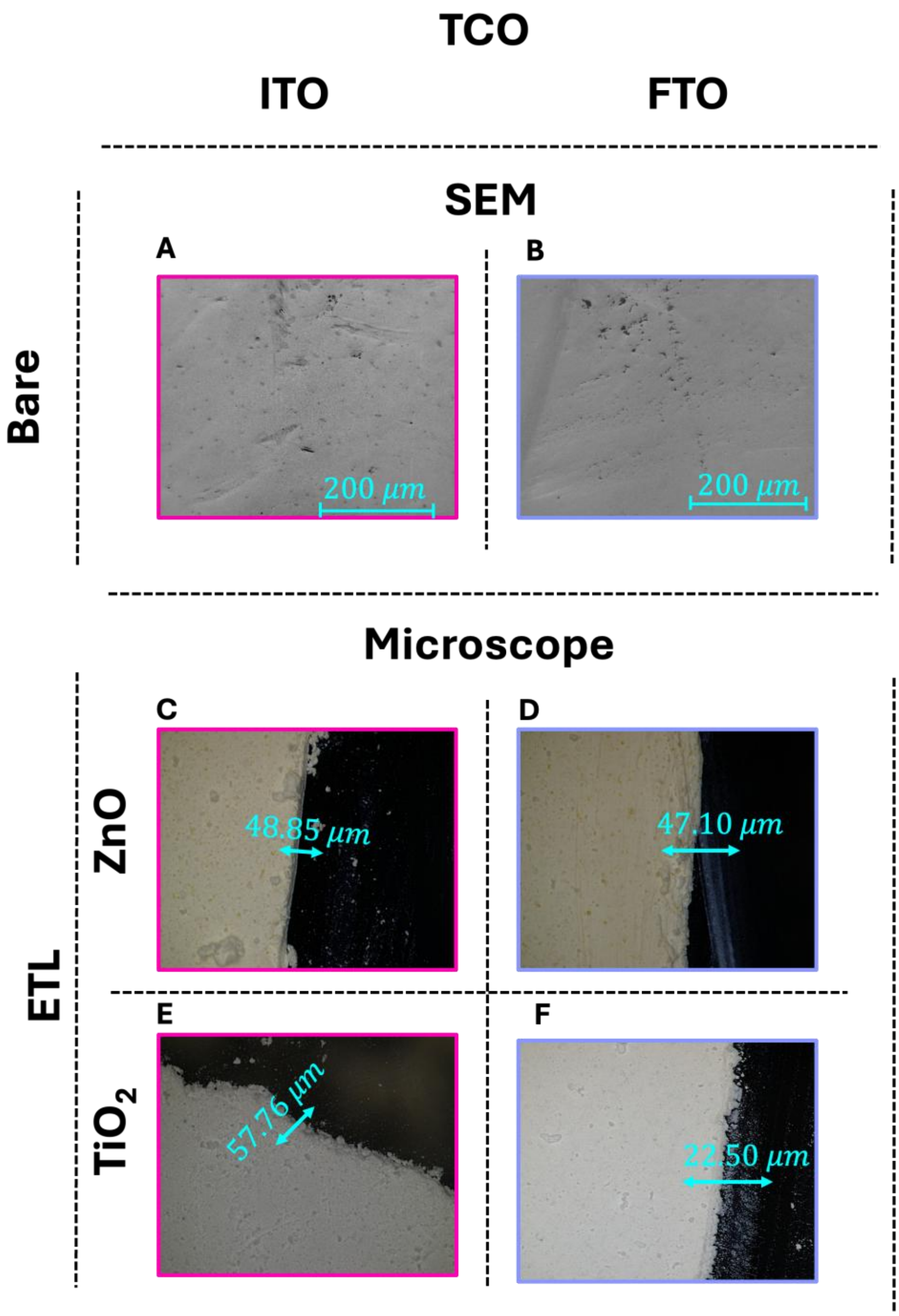

Figure S1. Morphological characterization of bare and oxide-coated ITO and FTO substrates. Scanning electron microscopy images of bare transparent conducting oxide substrates: **(A)** ITO and **(B)** FTO. Optical microscope cross-sectional images of deposited oxide layers on ITO and FTO: **(C)** ZnO@ITO, **(D)** ZnO@FTO, **(E)** TiO_2@ITO, and **(F)** TiO_2@FTO. The images show the surface morphology of the bare substrates and the approximate thickness/edge profile of the ZnO and TiO_2 coatings deposited on each transparent electrode. **Note:** These are local approximate thicknesses obtained at different positions and by different geometrical methods on spatially non-uniform drop-coated layers, rather than values expected to coincide exactly with the SEM averages.

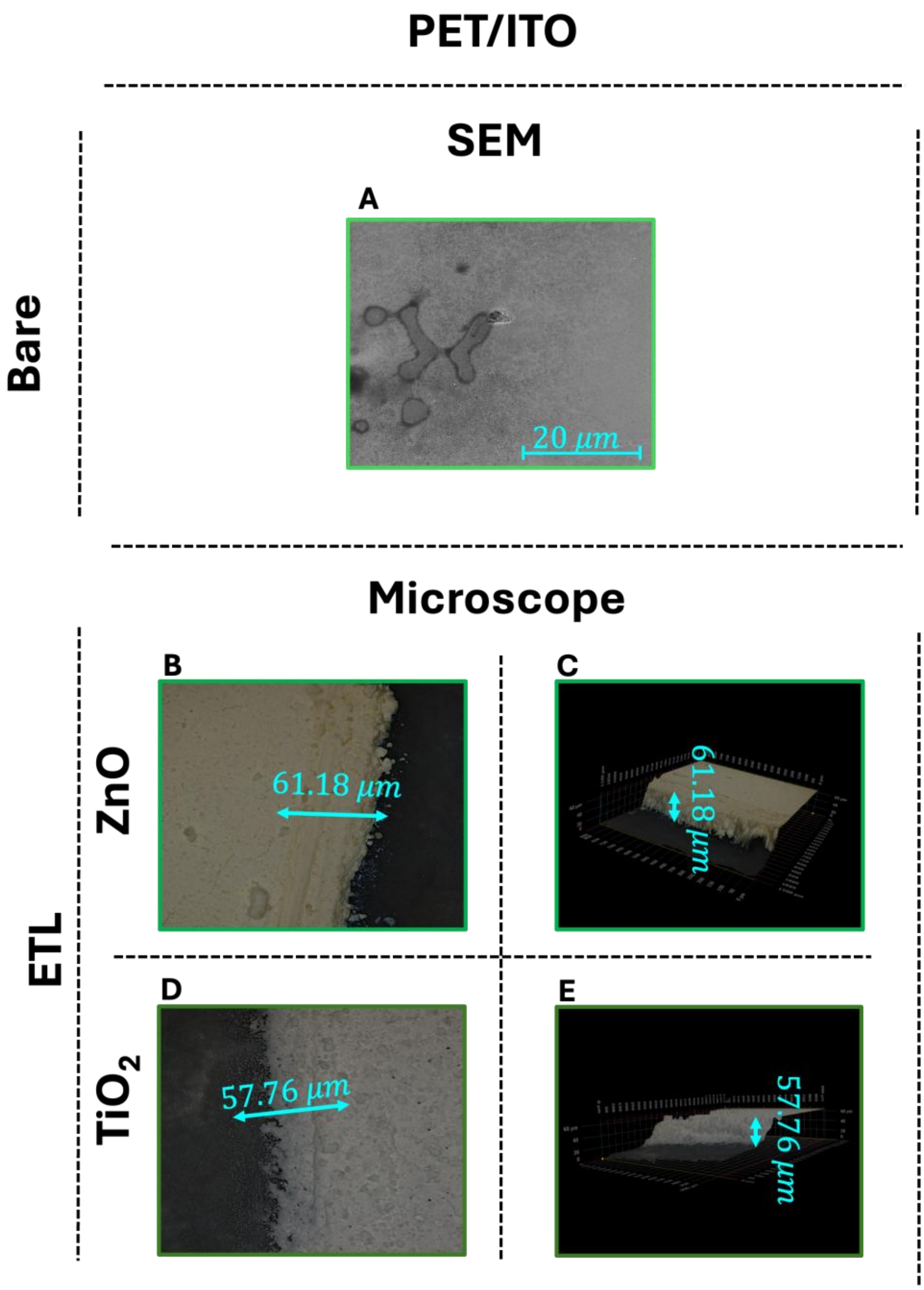

Figure S2. Morphological characterization of bare and oxide-coated PET/ITO substrates. **(A)** SEM image of the bare PET/ITO substrate. **(B,C)** Optical microscope image and corresponding three-dimensional surface reconstruction of the ZnO layer deposited on PET/ITO. **(D,E)** Optical microscope image and corresponding three-dimensional surface reconstruction of the TiO_2 layer deposited on PET/ITO. The measurements provide the approximate coating thickness and surface profile of the oxide layers on the flexible transparent conducting substrate.

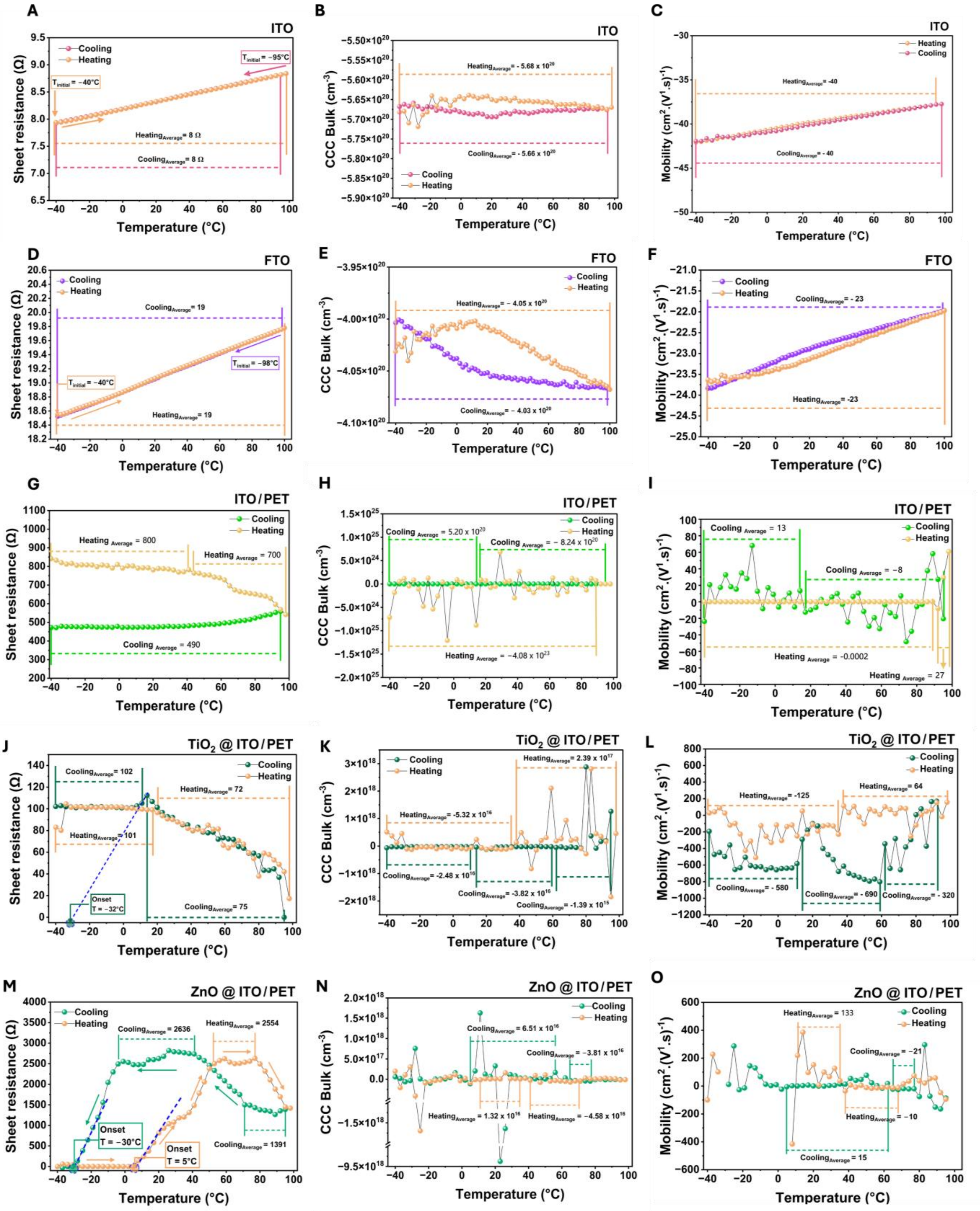

Figure S3. Temperature-dependent electrical properties of bare and oxide-coated transparent conducting substrates. Sheet resistance, charge carrier concentration, and carrier mobility measured during heating and cooling for **(A-C)** bare ITO, **(D-F)** FTO, **(G-I)** PET/ITO, and **(J-O)** oxide-coated PET/ITO systems. Panels include the electrical response of the bare transparent conducting oxides and the corresponding TiO_2- and ZnO-coated heterojunctions, allowing comparison of substrate-dominated and oxide-interface-dominated transport regimes.

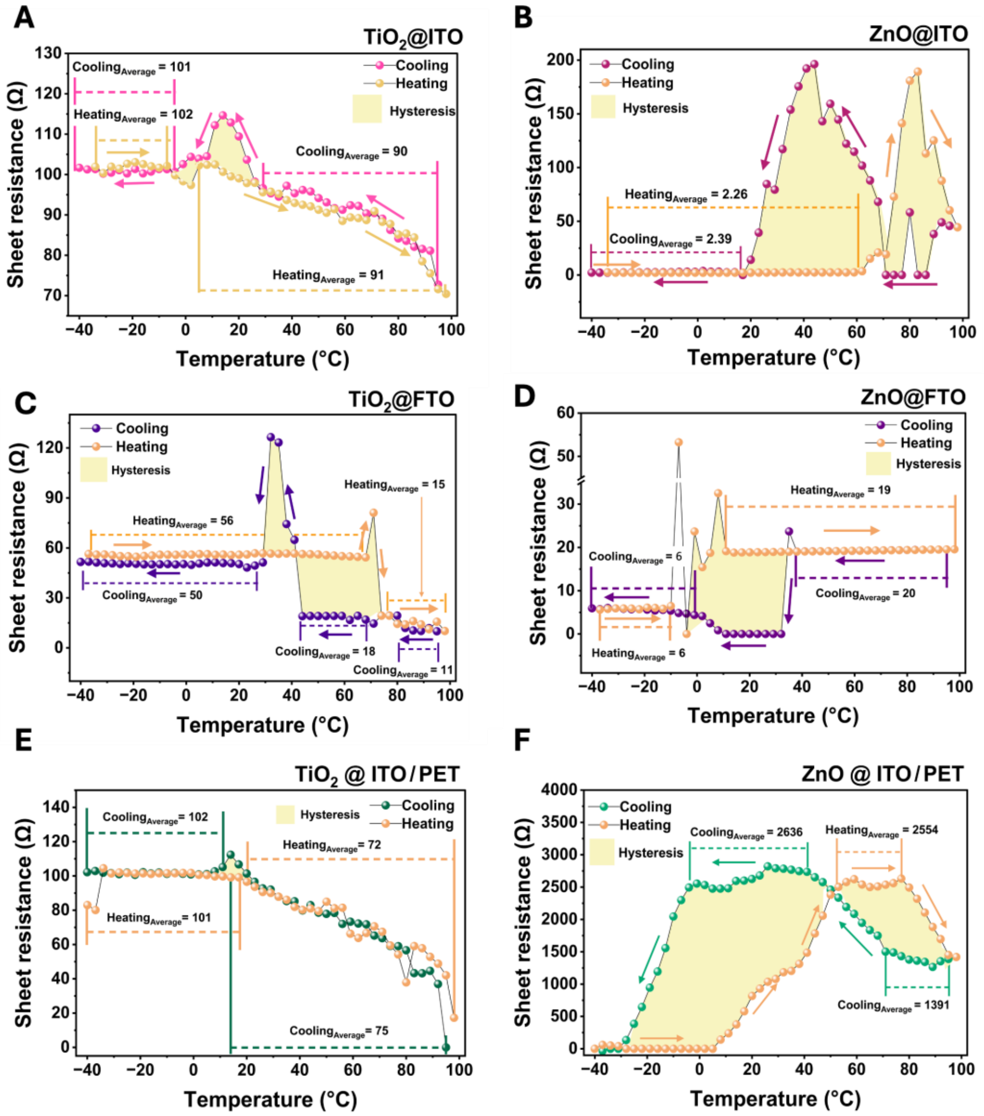

Figure S4. Thermal hysteresis in the sheet resistance of TiO_2- and ZnO-coated transparent conducting substrates. Temperature-dependent sheet resistance during heating and cooling for **(A)** TiO_2@ITO, **(B)** ZnO@ITO, **(C)** TiO_2@FTO, **(D)** ZnO@FTO, **(E)** TiO_2@PET/ITO, and **(F)** ZnO@PET/ITO. The shaded regions highlight the hysteretic behavior associated with thermally induced changes in the transport response of the oxide-coated heterojunctions.

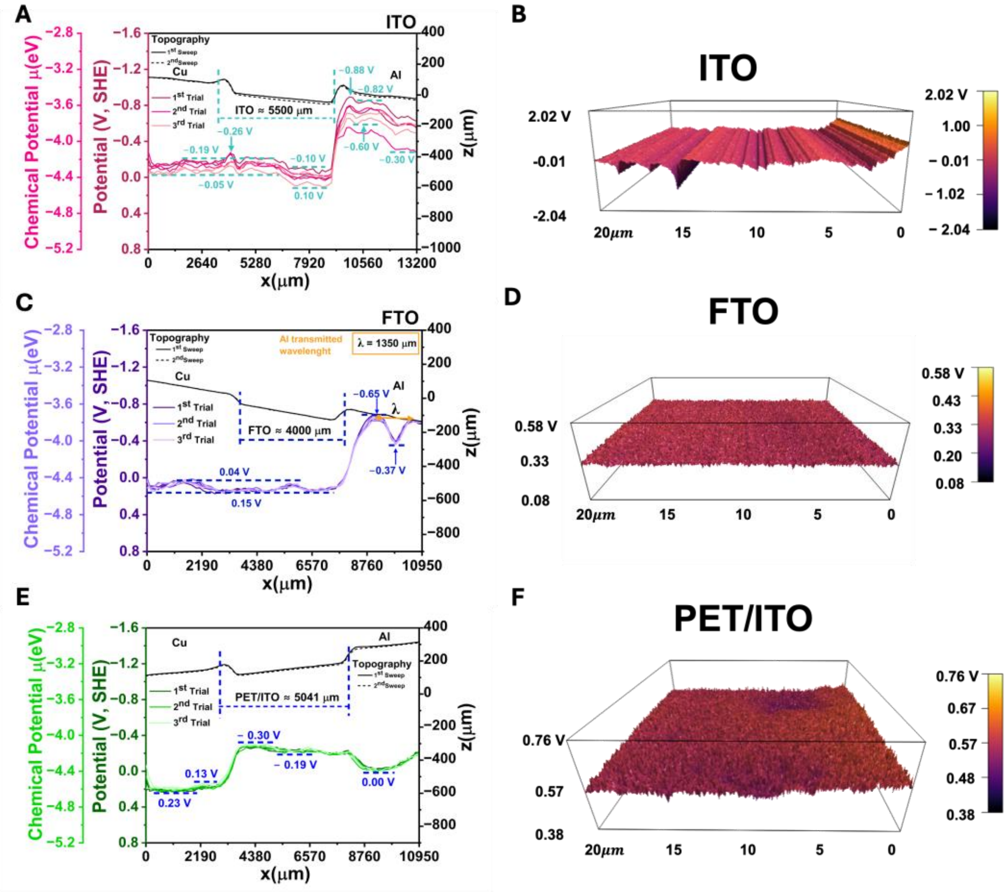

Figure S5. Surface-potential characterization of bare ITO, FTO, and PET/ITO substrates. Micrometric-resolution scanning Kelvin probe profiles and nanometric-resolution surface-potential maps of bare transparent conducting substrates. **(A,B)** ITO, **(C,D)** FTO, and **(E,F)** PET/ITO. The micrometric profiles were obtained in Cu/TCO/Al configurations, while the nanometric maps were acquired directly on the TCO surfaces, providing reference chemical-potential values for comparison with oxide-coated systems.

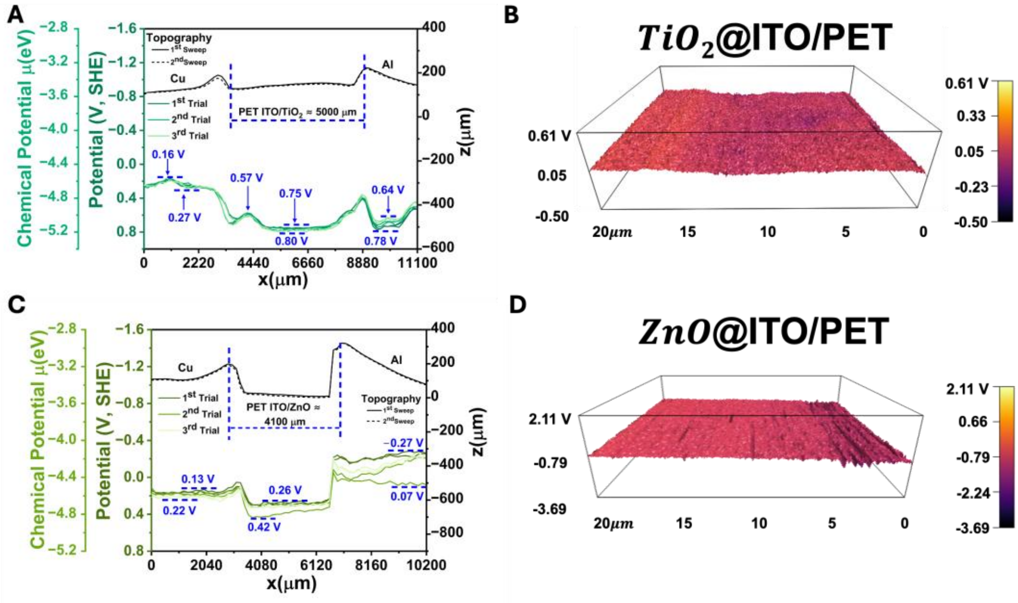

Figure S6. Surface-potential characterization of oxide-coated PET/ITO substrates. Micrometric-resolution scanning Kelvin probe profiles and nanometric-resolution surface-potential maps of oxide-coated PET/ITO heterojunctions. **(A,B)** TiO_2@PET/ITO and **(C,D)** ZnO@PET/ITO. The measurements compare the interfacial charge redistribution induced by TiO_2 and ZnO coatings on the flexible PET/ITO transparent electrode.

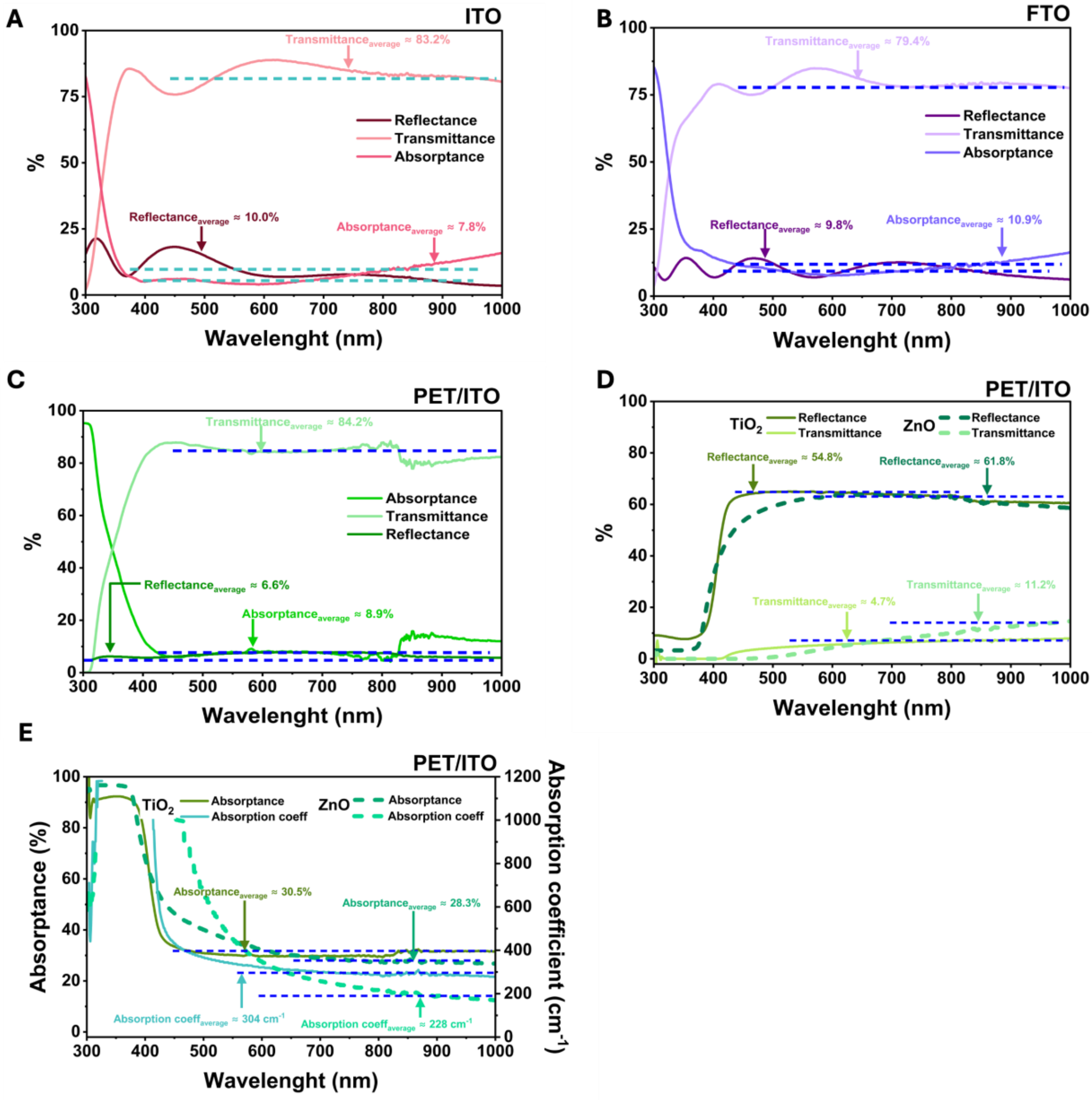

Figure S7. Optical response of bare and oxide-coated transparent conducting substrates. UV–visible reflectance, transmittance, and absorptance spectra of bare **(A)** ITO, **(B)** FTO, and **(C)** PET/ITO substrates. **(D)** Reflectance and transmittance spectra of TiO_2@PET/ITO and ZnO@PET/ITO. **(E)** Absorptance and absorption coefficient spectra of TiO_2@PET/ITO and ZnO@PET/ITO. These data complement the optical analysis of the glass-based ITO and FTO substrates by showing the behavior of the flexible PET/ITO platform. **Note:** Average thicknesses: PET/ITO; ITO thickness: 0.200 μm thick ITO layer; ZnO@PET/ITO: 61 μm; TiO_2@PET/ITO: 58 μm.
